# GPR120 prevents colorectal adenocarcinoma progression by sustaining the mucosal barrier integrity

**DOI:** 10.1038/s41598-021-03787-7

**Published:** 2022-01-10

**Authors:** Federica Rubbino, Valentina Garlatti, Valeria Garzarelli, Luca Massimino, Salvatore Spanò, Paolo Iadarola, Maddalena Cagnone, Martin Giera, Marieke Heijink, Simone Guglielmetti, Vincenzo Arena, Alberto Malesci, Luigi Laghi, Silvio Danese, Stefania Vetrano

**Affiliations:** 1 Laboratory of Molecular Gastroenterology, IRCCS Humanitas Research Hospital, Rozzano (Mi), Italy; 2 Department of Pharmaceutical Science, Università Degli Studi del Piemonte Orientale “Amedeo Avogadro”, Novara, Italy; 3 Institute of Nanotechnology, CNR NanoTec, Lecce, Italy; 4 Department of Biomedical Sciences, Humanitas University, Pieve Emanuele, Milan, Italy; 5 Department of Biology and Biotechnology “L. Spallanzani”, University of Pavia, Pavia, Italy; 6 Labanalysis L.L.C, Casanova Lonati, Pavia, Italy; 7 Center for Proteomics and Metabolomics, Leiden University Medical Center, Leiden, The Netherlands; 8 Division of Food Microbiology and Bioprocesses, Department of Food Environmental and Nutritional Sciences (DeFENS), Università Degli Studi Di Milano, Milan, Italy; 9 Fondazione Policlinico Universitario Agostino Gemelli, IRCCS Università Cattolica del Sacro Cuore, Rome, Italy; 10 Department of Medicine and Surgery, University of Parma, Parma, Italy; 11 Laboratory of Gastrointestinal Immunopathology, IBD Center, IRCCS Humanitas Research Hospital, Rozzano (Mi), Italy

**Keywords:** Cancer, Cell biology, Microbiology, Molecular biology, Gastroenterology

## Abstract

GPR120 (encoded by *FFAR4* gene) is a receptor for long chain fatty acids, activated by ω-3 Polyunsaturated Fatty Acids (PUFAs), and expressed in many cell types. Its role in the context of colorectal cancer (CRC) is still puzzling with many controversial evidences. Here, we explored the involvement of epithelial GPR120 in the CRC development. Both in vitro and in vivo experiments were conducted to mimic the conditional deletion of the receptor from gut epithelium. Intestinal permeability and integrity of mucus layer were assessed by using Evans blue dye and immunofluorescence for MUC-2 protein, respectively. Microbiota composition, presence of lipid mediators and short chain fatty acids were analyzed in the stools of conditional GPR120 and wild type (WT) mice. Incidence and grade of tumors were evaluated in all groups of mice before and after colitis-associated cancer. Finally, GPR120 expression was analyzed in 9 human normal tissues, 9 adenomas, and 17 primary adenocarcinomas. Our work for the first time highlights the role of the receptor in the progression of colorectal cancer. We observed that the loss of epithelial GPR120 in the gut results into increased intestinal permeability, microbiota translocation and dysbiosis, which turns into hyperproliferation of epithelial cells, likely through the activation of β -catenin signaling. Therefore, the loss of GPR120 represents an early event of CRC, but avoid its progression as invasive cancer. these results demonstrate that the epithelial GPR120 receptor is essential to maintain the mucosal barrier integrity and to prevent CRC developing. Therefore, our data pave the way to GPR120 as an useful marker for the phenotypic characterization of CRC lesions and as new potential target for CRC prevention.

## Introduction

G protein-couple receptors (GPCRs) are responsible for the signal transduction of a wide variety of ligands, including hormones, growth factors, and lipids^1,2^ mediating several cellular processes, and so have great potential as therapeutic targets for a broad spectrum of diseases including tumor^3^. GPR120, also named free fatty acid (FAs) receptor-4 (*FFAR4*), the receptor for long-chain fatty acids, activated by n3 Polyunsaturated Fatty Acids (ω-3 PUFA)^4,5^, mediates insulin sensitizing and anti-diabetic effects by repressing macrophage-induced tissue inflammation playing a key role in metabolic and inflammatory diseases. However, GPR120 is expressed also in several cell types, including the intestinal epithelial cells^6,7^, where seems to exert anti-inflammatory effects once activated by PUFAs^4,8^. In tumors, GPR120 expression positively correlated with clinical response to chemotherapy in patient with breast cancer^9^ and its activation promotes metastasis in lung tissue through PI3K/Akt/NF-KB signaling^10^. Increased level is detected in esophageal cancer, in which its expression positively correlates with tumor progression^11^. In prostate cancer cell-lines, GPR120 promotes the anti-cancer effects of ω-3 PUFAs by blocking ERK1/2 signaling pathway and cell proliferation, migration and survival^12^. On the other side, activation of GPR120 by selective agonists may exacerbate the malignant phenotype of pancreatic cancer cell lines^13^. Its expression positively correlated with advanced tumor stage in colorectal cancer (CRC) patients^14^. Although some evidences showed that GPR120 enhances angiogenesis and dissemination promoting epithelial-to-mesenchymal transition (EMT)^15^, GPR120 function in colorectal cancer development is still puzzling.

Despite the advances in screening and surveillance, CRC remains the second most common cause of cancer death worldwide for which the molecular mechanisms driving the disease are still not clear. To date, is important to identify new potential molecules for tumor-preventive strategies. Here, we highlighted the functional role of GPR120 in the epithelial compartment and its involvement in the intestinal tumorigenesis providing evidence that the receptor contributes to the integrity of intestinal epithelial barrier. This barrier system, while avoiding harmful penetration, is selectively permeable to allow absorption of water and nutrients^16^. Thus, on the one hand it prevents the attachment and entry of pathogens into intestinal mucosa, and other hand cross-talks with the commensal microflora, which in turn contributes to the barrier by producing hydrosoluble and liposoluble vitamins [vitamin K, folate,] and short chain fatty acids (SCFAs)^17^. Emerging data demonstrate that defects of barrier function, as well as reduced mucus production, are early biological events in colorectal tumorigenesis, being crucial for the activation of underlying immune cells by microbial products^18–20^. We observed that GPR120 plays an important role in controlling intestinal homeostasis and restraining tumor development. Our data highlight new activities exerted by GPR120 in CRC progression, and point at its potential role as a therapeutic target for CRC prevention.

## Results

### GPR120 is mainly expressed in gut epithelial cells

**Figure 1:**
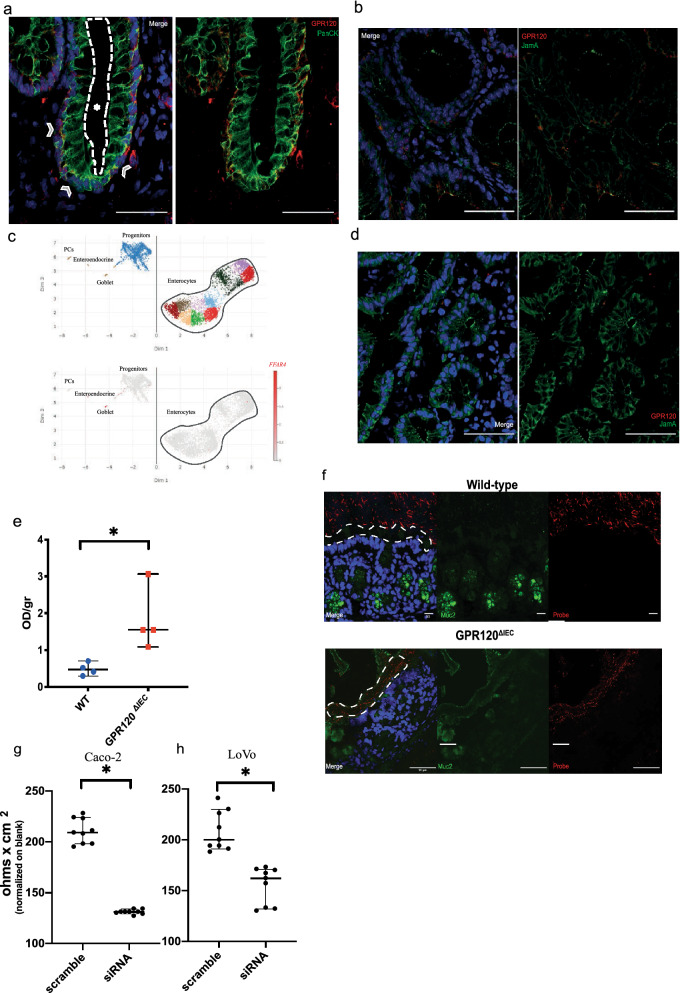
GPR120 expression in intestinal epithelial cells. **(a, b**) Representative immunofluorescence images from the human (**a**) and mouse (**b**) frozen colon sections stained with antibodies against GPR120, cytokeratins (Pan CK) and junctional adhesion molecule A (Jam A). In the panel (a), arrows indicate the basolateral side of the epithelium, while the star (asterisk) is in the lumen and is delimited by dashed line. Magnification: 40X. Scale bar: 50 μm. (**c**) Reanalysis of single-cell RNA-seq data from human epithelial cells (GEO ID GSE125970). Cell dispersion within the UMAP multidimensional scaling space, colored by cluster or by *FFAR4* normalized expression, and labeled by cell type. *FFAR4* is clearly expressed by Goblet cells and a subset of progenitors. (**d**) Representative immunofluorescence images from frozen colon tissues of transgenic (GPR120^ΔIEC^) mice stained with antibodies against GPR120 and Jam A. Magnification: 20x. Scale bar: 50 μm. (**e)** Intestinal permeability was evaluated in healthy GPR120 ^ΔIEC^ mice (n = 4) and WT (n = 4) by perfusion in the mouse intestine with Evans Blue dye for 60 min. The amount of dye eluted was quantified using a spectrophotometer at a wavelength of 620 nm and results are expressed as OD per gram of colon tissue. (**f**) Frozen colon sections from healthy GPR120 ^ΔIEC^ mice (n = 3) and WT (n = 3) littermates were stained with FISH using a bacterial probe (red) and an anti-Muc2 antibody (green). The inner mucus layer is indicated with white dashed line. Magnification 40x. Scale bar: 50 μm. (**g-h**) TEER was measured in siRNA-treated Caco-2 and LoVo cells and compared with scramble siRNA controls**.** Values are expressed as median ± 95% CI. *p value < 0,05; **p.value < 0,001 by Mann Whitney test.

Accordingly with other data^21^, GPR120 is expressed by intestinal epithelial cells as demonstrated by the co-localization of the receptor with epithelial markers, such as cytokeratin (Fig. 1a) and junctional adhesion molecule A (Jam A) (Fig. 1b). To determine its expression in all epithelial cells, we performed an in silico analysis on epithelial single-cell RNA-seq data^22^ (GSE125970). As reported in Fig. 1c, *FFAR4* gene is mainly expressed in goblet cells and subset of progenitors. Accordingly, the immunostaining revealed a strong positivity for GPR120 in the cells cup-like appearance, typical goblet cell phenotype, as evidenced in Supplementary Fig. S1a.

### GPR120 is involved in the maintenance of mucosal barrier integrity

To explore the functional role of GPR120 on epithelial compartment, we generated mice with a conditional deletion of GPR120 (named here GPR120^ΔIEC^) on epithelial cells crossing GPR120^flox/flox^ mice with *VillinCre* mice^23^. Transgenic mice did not display any evident phenotype in terms of body weight, overall survival, colon and small intestine length in healthy condition, compared to WT littermates. The GPR120 knockdown efficiency in the epithelial compartment was checked by immunofluorescence on colon tissues from both WT and GPR120^ΔIEC^ animals (Fig. 1b,d) and in small intestine by immunohistochemistry (Supplementary Fig. S1b).

**Figure 2:**
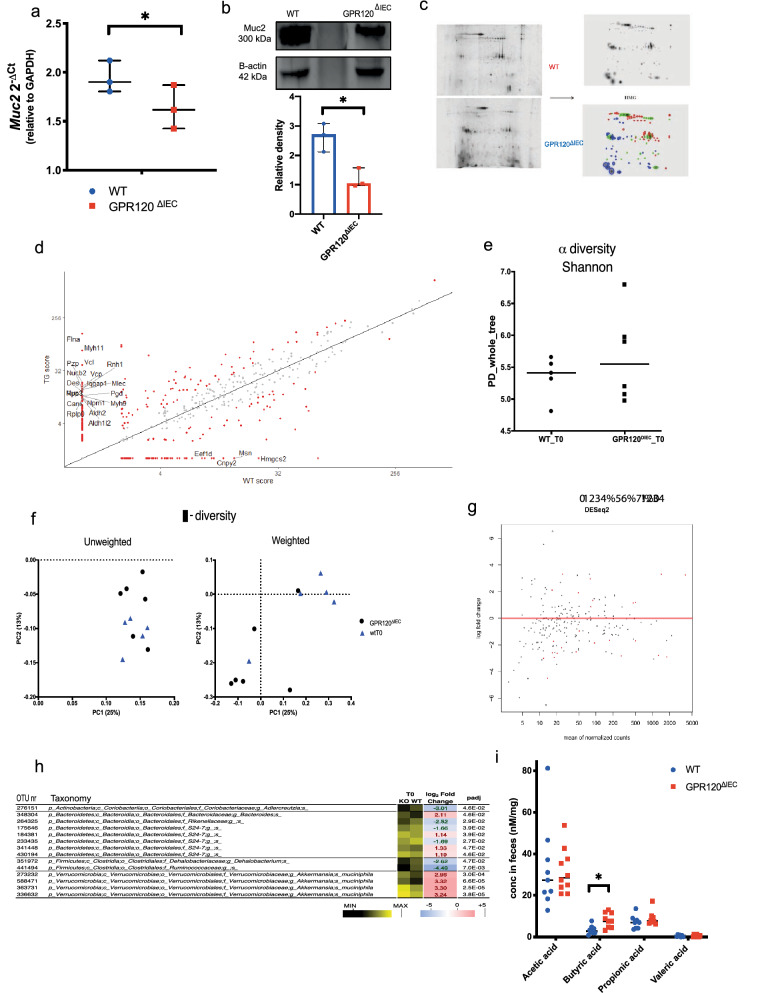
GPR120 expression preserves inner mucus layer integrity. **(a)** The expression levels of *Muc2* gene was quantified by qRT-PCR in healthy GPR120^ΔIEC^(n = 3) and WT (n = 3) mice. (**b**) Upper panel shows a representative band of Muc2 obtained by western blot on GPR120^ΔIEC^ and WT mucus samples. The relative density of these bands is reported in the lower panel (n = 3). (**c)** Mucus layer samples from healthy GPR120^ΔIEC^ (n = 3) and WT (n = 3) mice were scraped off the colon and subjected to two-dimensional electrophoresis. Gels from transgenic and WT mucus were overlapped in a high master gel (HMG) by software to identify proteins differentially expressed in the two groups. Red spots belong to WT samples, blue spots belong to GPR120^ΔIEC^ samples, and green spots are in common between two groups. (**d)** Scatter plot showing all proteins detected by mass-spectrometry and differentially expressed (in red) between GPR120^ΔIEC^(TG) and WT mice, according to DAVE score + 2/-2. Among dysregulated proteins, Muc2 was significantly downregulated in GPR120^ΔIEC^ mucus samples. (**e**) A metagenomic analysis was performed on fecal samples of healthy WT (n = 5) and GPR120 ^ΔIEC^ (n = 6) mice. Plot showing alpha diversity (Shannon index) for microbial communities in the two groups, upon16S rRNA gene sequencing analysis. (**f)** Principal component analysis (PCA) plots showing Weighted (right panel) and Unweighted (left panel) beta diversity in fecal samples from the two groups. (**g)** DESeq2 analysis showing operational taxonomical units (OTUs) in GPR120 ^ΔIEC^ versus WT fecal samples. Each spot represents a taxonomical unit; red spots represent taxonomical units with statistically significant differences between the two groups. (**h**) OTUs distinguishing WT and GPR120 ^ΔIEC^ mice determined using the DESeq2 negative binomial distribution method on the 16S rRNA gene profiling data of fecal samples. The taxonomic lineage of each taxon is shown: p, phylum; c, class; o, order; f, family; g, genus; s, species. The black-yellow heatmap represents the mean normalized relative abundances of the reported OTUs. Positive fold changes (shown on a red background) designate OTU overrepresentation in GPR120 ^ΔIEC^ mice (KO); negative fold changes (shown on a blue background) designate the OTU overrepresentation in WT mice. padj, adjusted p values were represented in a heatmap. (**i**) Bar plots showing quantification of SCFAs, including acetate, butyrate, propionate and valerate in fecal samples of healthy GPR120^ΔIEC^ (n = 10) and WT (n = 9) littermates. Values are expressed as median ± 95% CI. *p.value < 0.05 by Mann Whitney test.

Since GPR120 is highly expressed in goblet cells, specialized epithelial cells with well-appreciated role in barrier maintenance through the secretion of mucus, we next explored the involvement of GPR120 in maintaining the integrity of mucosal barrier. To achieve this aim, we evaluated the intestinal permeability by perfusing the colonic mucosa of healthy GPR120^ΔIEC^ and WT littermates with Evans blue dye^24^. The assay revealed a significant increased dissemination of the dye into the mucosa of GPR120^ΔIEC^ compared to WT littermates (Fig. 1e). This impairment was further confirmed by fluorescence in situ hybridization (FISH) of the colonic mucosa showing bacteria able to penetrate the MUC2 mucin-dependent mucus layer only of GPR120^ΔIEC^ mice (Fig. 1f). Interestingly, the expression analysis of *MUC2* resulted significantly reduced in healthy transgenic mice at gene and protein levels (Fig. 2a,b) providing a clear evidence that GPR120 is involved in regulating permeability. To gain insight into the mechanisms underlying this function, we modulated the expression of GPR120 receptor in vitro in epithelial CRC cell lines Caco-2 cells and LoVo, both expressing high levels of GPR120 among the six cell lines analyzed (Supplementary Fig. S1c).

After silencing of GPR120 using a specific siRNA (Supplementary Fig. 1d), both cell lines were subjected to Transepithelial Electrical Resistance (TEER) measurement, showing a significant reduced resistance in both siRNA-treated cell lines (Fig. 1g,h) than scramble controls, thus corroborating a role for GPR120 in maintaining mucosal barrier integrity. In addition, a preliminary analysis performed on primary epithelial cells lacking of GPR120, revealed a significant down-regulation of genes encoding for basement membrane proteins (Supplementary Fig. S1e).

### Epithelial GPR120 deletion affects mucus and microbiota composition

Since MUC2 expression was impaired in healthy GPR120 ^ΔIEC^ mice (Fig. 2a,b)—but not in tumor bearing mice (Supplementary Fig. S1f)—we analyzed the protein composition of the mucus layer. The reconstruction of GPR120^ΔIEC^ and WT mucus samples, allowed the identification of 117 spots representing different proteins. Out of total spots, 29 were shared between GPR120^ΔIEC^ and WT mice, whereas 48 were detectable uniquely in WT and 40 uniquely in GPR120^ΔIEC^ mucus samples (Fig. 2c). Correspondingly, we found clear differences in the mucus composition between the two experimental groups (Fig. 2d). Among the proteins detected only in GPR120^ΔIEC^ mucus samples, Myosin 11 (Myh11), Myosin 9 (Myh9), and Vinculin (Vcl) which are related to mucosal barrier integrity were identified, strengthening the finding that the loss of the receptor in the intestinal epithelium affects mucus composition.

Next, we performed a microbiome analysis by 16S rRNA gene profiling on faecal samples collected from healthy GPR120^ΔIEC^ and WT mice, in which no differences were detected in alpha- and beta-diversity comparisons between two groups (Fig. 2e,f). A differential expression analysis based on the negative binomial distribution (DESeq2) allowed us to identify all differentially expressed operational taxonomic units (OTUs), with or without statistical significance (Fig. 2g). Based on the taxonomic results, we found differences in the microbiota composition of GPR120^ΔIEC^ and WT mice even at steady state. In fact, we found 14 OTUs whose normalized relative abundance was significantly different between the two groups of mice (Fig. 2g). In specific, 8 OTUs were overrepresented in mutant mice, including taxonomic units ascribed to the genus *Bacteroides* (1 OTU), the family S24-7 (3 OTUs), and, particularly, to the species *Akkermansia muciniphila* (4 OTUs) (Fig. 2h). Conversely, 6 OTUs were overrepresented in WT mice, including taxonomic units ascribed to the genera *Adlercreutzia* and *Dehalobacterium*, and the families *Rikenellaceae*, S24-7 and *Ruminococcaceae* (Fig. 2h). While, in tumor-bearing mice, most of the observed differences between transgenic and control mice are referred to the OTUs ascribed to the *Bacteriodales family,* decrease in *Firmicutes,* and concomitant expansion of *Proteobacteria* (Supplementary Fig. S2a). A fundamental role of microbiota is the production of SCFAs, for their trophic effects on intestinal epithelium^17^. Among the four major SCFAs produced as microbial metabolites—acetate, propionate, butyrate and valeric acid—our results showed that only butyric acid was significantly upregulated in transgenic mice in healthy condition (Fig. 2i).

### GPR120 attenuates tumorigenesis during inflammation-driven colorectal cancer

**Figure 3:**
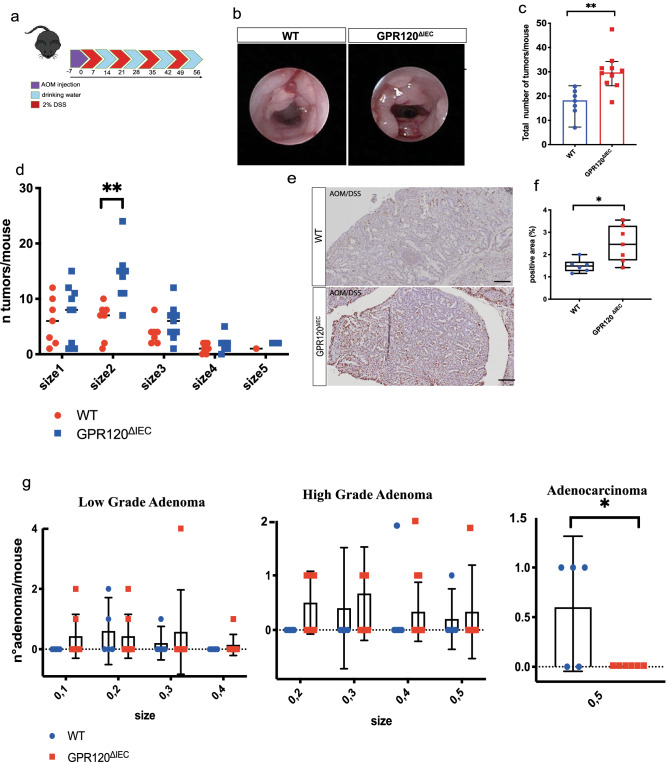
Conditional deletion of GPR120 in the intestinal epithelium affects colitis-associated cancer development. (**a**) GPR120^ΔIEC^ (n = 7) and WT (n = 7) mice were subjected to the AOM/DSS-induced model of CRC as schematize. (**b**) Representative endoscopic images showing mouse polyps at day 57. Tumor density (**c**) and size (**d**) assessed at day 57 by endoscopic scoring. The size depends upon the average of diameter: size 1 very small but detectable tumor, size 2 tumor covering up to one-eighth of colonic circumference, size 3 tumor covering up to one-fourth of the colonic circumference, size 4 tumor covering up to half of the colonic circumference, and size 5 tumor covering more than half of the colonic circumference. (**e, f**) Representative paraffin-embedded colon sections from tumor bearing GPR120^ΔIEC^ (n = 7) and WT (n = 7) mice stained for Ki67 (**e**) and relative quantification expressed as percentage of immune positive area (**f**). Magnification 20x. Scale bar: 100 μm. (**g**) Histological score quantifying Low grade (left panel), High grade (central panel) adenomas, and adenocarcinoma (right panel) on paraffin-embedded colon sections (n = 7 per group) stained with Hematoxylin & Eosin (H&E).

Emerging data demonstrate that defects of barrier function, as well as reduced mucus production, are early biological events in colorectal tumorigenesis. Hence, it was important to investigate whether GPR120 deficiency leads to more severe colitis-associated cancer (CAC). To verify that, GPR120^ΔIEC^ and WT mice were subjected to the azoxymethane (AOM)/dextran sodium sulfate (DSS)-induced model of colitis-associated CRC (Fig. 3a). Results revealed no significant differences in clinical signs of intestinal inflammation between GPR120^ΔIEC^ and WT mice, as shown by comparable body weight loss (Supplementary Fig. S2b), and DAI score (Supplementary Fig. S2c), throughout the entire experiment, as well as in genes encoding for pro-inflammatory cytokines like IL6, IL1ß and TNFα (Supplementary Fig. S2d).

Tumor growth was quantified by colonoscopy at the termination end point (Fig. 3b) as described previously^25^. Transgenic mice showed a significant increase of tumor density along the colon in comparison to WT animals (Fig. 3c). Furthermore, the histological analysis of the colon tissue revealed that tumor lesions in GPR120^ΔIEC^ mice were characterized by a higher number of small-sized tumors (Fig. 3d). This finding was consistent with a significantly higher number of proliferating cells in colons from GPR120^ΔIEC^ mice, as shown by immunohistochemical staining for the proliferative marker Ki67 (Fig. 3e,f). While both wild-type and transgenic mice developed low- and-high grade adenomas (Fig. 3g, left and center panels), strikingly GPR120 ^ΔIEC^ mice did never develop any adenocarcinoma (Fig. 3g, right panel). This finding suggests that epithelial GPR120 is crucial in the early event of dysplastic lesions rather than progression.

### GPR120 expression is reduced in human adenocarcinoma

**Figure 4:**
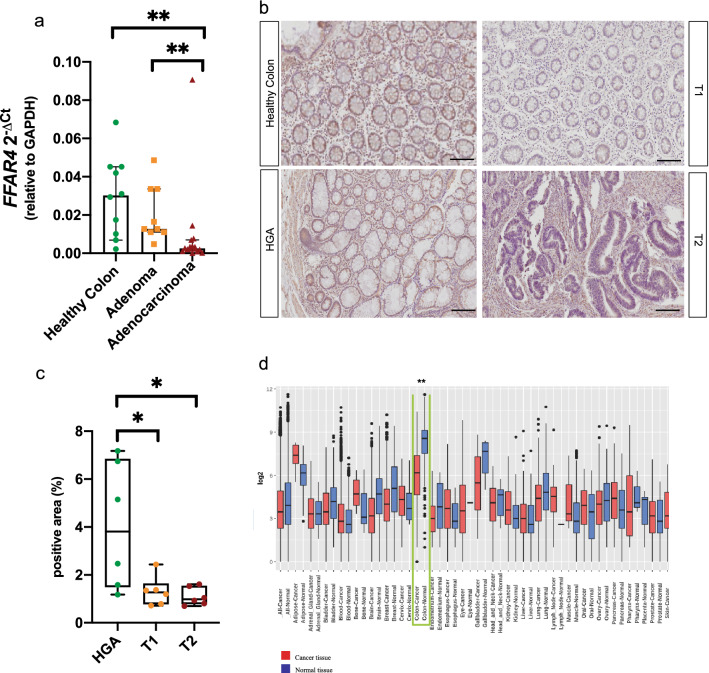
Loss of epithelial GPR120 correlates with CRC development (**a**) The expression levels of *FFAR4* gene were quantified by qRT-PCR in healthy colon (n = 9), adenomas (n = 9) and adenocarcinoma (n = 17) from human samples. (**b**) Representative immunostaining for GPR120 in human samples of Normal mucosa (n = 6), High-Grade Adenomas (HGA) (n = 6), T1 (n = 6) and T2 (n = 6) tumors, (**c**) and relative quantification of positive area. Values are expressed as median percentage of positive over total area ± 95% CI. Magnification: 20X*.* Scale bar: 50 μm. (**d**) Gene expression of *FFAR4* in the indicated tissues annotated in the GENT2 database, expressed as Log2 and plotted according to tissue type. Values are expressed as median (horizontal line) ± IQR (interquartile range, boxes), dots represent outliers. Green box highlights *FFAR4* expression in CRC (n = 4047) and healthy (n = 398) colon tissue. *p value < 0.05; **p.value < 0.001 by Mann Whitney test (a-c) and Student *t* test (**d**).

Given the loss of epithelial GPR120 results in increased adenoma incidence in mouse model, we explored its expression in a retrospective cohort of human adenomas and adenocarcinomas. We found that the expression of *FFAR4* gene decreases increasing the grade of the lesion (Fig. 4a). More specifically, the expression of the receptor is significantly downregulated in adenocarcinomas compared to healthy colon, as well as compared to adenomas. This was confirmed at protein level by immunostaining (Fig. 4b,c), in which the expression of GPR120 was significantly higher in High Grade Adenoma (HGA), compared to stage 1 and 2 tumors (T1 and T2). Importantly, GPR120 expression was drastically reduced in the epithelium (Supplementary Fig. S3) and detectable on immune infiltrate. Additionally, in silico analysis performed by using Gene Expression Across Normal and Tumor tissue (GENT2) database, revealed *FFAR4* significantly downregulated in CRC tissues when compared to healthy controls (Fig. 4d). All these human evidences support the hypothesis that epithelial GPR120 is not only involved in the early stage of CRC, but it also has a role in the progression*.*

### GPR120 modulation impacts epithelial cell proliferation

**Figure 5:**
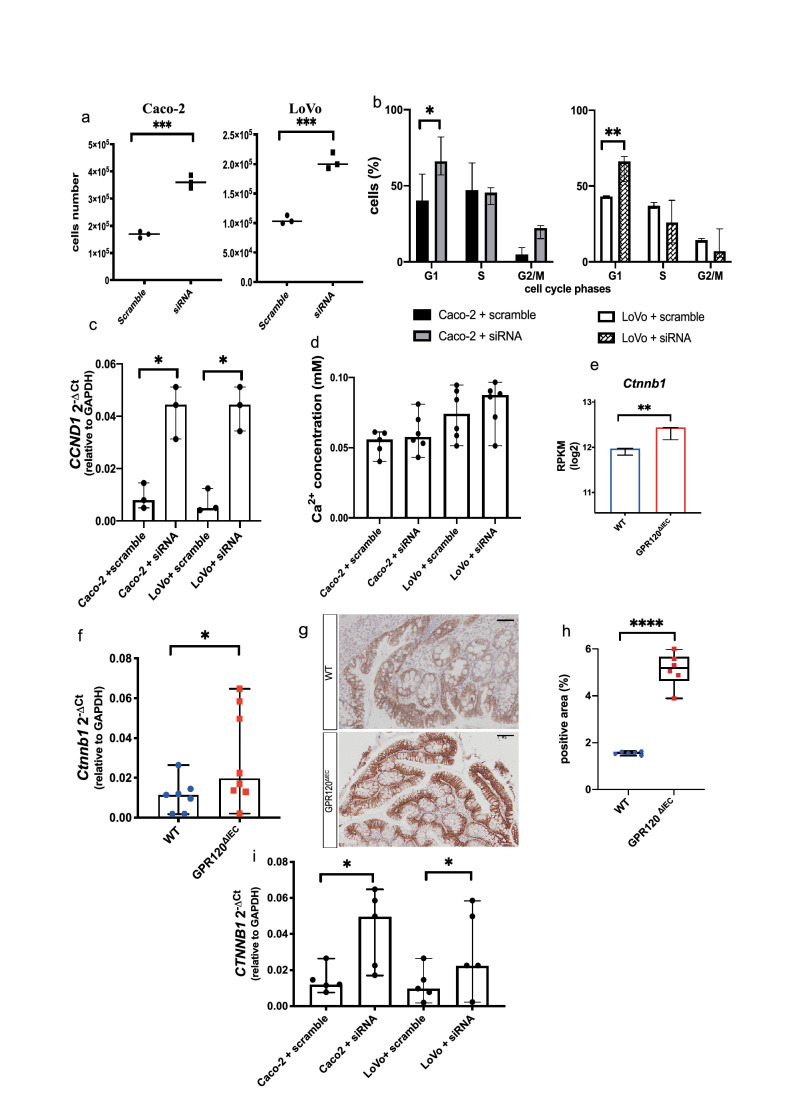
GPR 120 protein induces Wnt/β-catenin signaling. (a) Caco-2 (left panel) and LoVo (right panel) cells were transfected with siRNA against *FFAR4* and scramble siRNA controls, after 48 h from transfection proliferation was assessed by using the Burker chamber and expressed as absolute cell number. (**b**) The cell cycle distribution of siRNA-treated Caco-2 and LoVo cells was performed by FACS 48 h after siRNA transfection, and upon DAPI staining. Quantifications of Caco-2 and LoVo cells labeled with DAPI in each phase of the cell cycle are shown. Values are median ± 95% CI of biological triplicates. (**c**) Quantification of *CCND1* gene transcripts by qRT-PCR in Caco-2 and LoVo cells treated with either siRNA against *FFAR4* or scramble siRNA. (**d**) Intracellular calcium was measured 48 h after siRNA transfection, by using a colorimetric kit and results are expressed as mM of calcium concentration. Biological triplicates. (**e**) The expression of β-catenin gene transcript (*Ctnnb1*) was extrapolated from our RNA-seq analysis performed on the epithelium of healthy GPR120 ^ΔIEC^ (n = 3) and WT (n = 3) mice, and expressed as log2 RPKM. (**f**) *Ctnnb1*expression levels were quantified by qRT-PCR in AOM/DSS-induced GPR120 ^ΔIEC^ (n = 7) and WT (n = 7) mice. (**g,h**) Paraffin-embedded colon sections from GPR120 ^ΔIEC^ (n = 7) and WT (n = 7) mice were immunostained using an anti-β-catenin antibody. Magnification: 20X. Scale bar: 100 μm. Representative immunostainings are shown in (**g**), with relative quantification of immune-positive areas in (**h**). Values are expressed as median percentage of positive over total area ± 95% CI. (**i**) Quantification of *CTNNB1* gene transcripts by qRT-PCR in Caco-2 and LoVo cells treated with either siRNA against *FFAR4* or scramble siRNA. Values are expressed as median ± 95% CI of biological triplicates. *p.value < 0.05; **p.value < 0.001; ****p.value < 0.00005 by Mann Whitney test.

To better investigate the role of GPR120 in epithelial cell proliferation (Fig. 3e,f), we explored the effects of GPR120 silencing on human cancer cell lines. Both siRNA-treated Caco2 and LoVo cells displayed an increased number of proliferative cells in comparison with their relative controls (Fig. 5a, left and right panel respectively). Then, we performed both cell cycle analysis and apoptosis detection on siRNA-treated and scramble control cells. Both Caco-2 and LoVo siRNA silenced cells showed a higher percentage (30% more) of cells in G1 phase (Fig. 5b, left and right panel respectively), while the percentage of apoptotic cells was comparable in both groups (Supplementary Fig. S4a,b). Of note, the gene encoding for Cyclin D1, promoter for G1-to-S transition, was even significantly up-regulated in siRNA treated cells (Fig. 5c). As some evidences reported that GPR120 regulates cell proliferation modulating intracellular levels of Ca^2+^^26,27^, we measured its levels after GPR120 silencing. Results showed no differences in the concentration of intracellular Ca^2+^ between siRNA-treated Caco-2 and LoVo cells and their relative scramble siRNA-treated controls (Fig. 5d), indicating that GPR120 does not affect proliferation of CRC cells by modulation of Ca^2+^ influx. From our functional enrichment analysis on DEGs in the RNAseq, performed on epithelial cells isolated from healthy wild-type and transgenic mice, we observed biological processes related to Wnt pathway signaling to be the most significantly enriched in GPR120^ΔIEC^ compared to WT epithelium. This pathway is known to be involved in cell growth, differentiation, and apoptosis and one of its key mediators is represented by β-catenin^28^.

A deeper analysis showed indeed a significative upregulation of *Ctnnb1,* the gene encoding for β-catenin, in the epithelium of GPR120^ΔIEC^ healthy mice compared to WT (Fig. 5e). Interestingly, this data was confirmed not only in tumor bearing GPR120^ΔIEC^ animals versus WT, as shown by qRT-PCR (Fig. 5f), and immunohistochemical analysis on colon sections from AOM/DSS-treated mice (Fig. 5g,h), but also on Caco-2 and LoVo cells, upon GPR120 silencing (Fig. 5i), suggesting that GPR120 may directly affect CRC cell proliferation through β-catenin signaling.

### Epithelial GPR120 influences the fecal lipidomic profile of AOM/DSS treated mice

**Figure 6:**
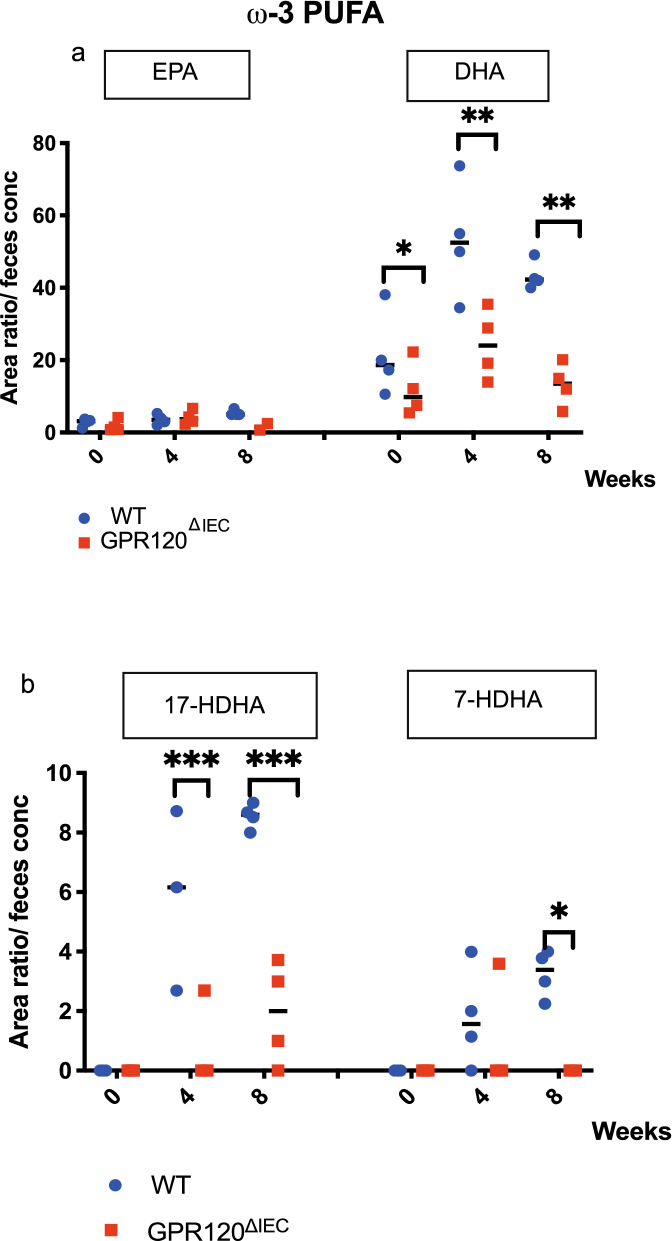
The loss of epithelial GPR120 impacts on fecal lipids metabolites. (**a,b**) ω-3 PUFAs (**a**) and DHA-derived metabolites (**b**) were quantified through LC–MS/MS analysis and expressed as area ratio/feces concentration. Values are expressed as median ± 95% CI. *p.value < 0.05; **p.value < 0.001; ****p.value < 0.00005 by Mann Whitney test.

Since the ω-3 polyunsaturated fatty acids (PUFAs), docosahexaenoic acid (DHA) and eicosapentaenoic acid (EPA) are established activators of GPR120^5^, and a current body of literature supports their beneficial effects in the context of CRC, we analyzed the lipidomic profile in healthy and tumor-bearing mice. Results showed that while no differences were evident in the amount of ω-6 PUFAs between GPR120 ^ΔIEC^ and WT mice at any time point (Supplementary Fig. S5a), among ω-3 PUFAs a significant downregulation of DHA was apparent at each time point in GPR120^ΔIEC^ mice compared to WT (Fig. 6a). Among DHA-derived lipid mediators, we found a significant reduction of 17-HDHA both at 4 and 8 weeks in transgenic mice versus WT (Fig. 6b), and the 7-HDHA downregulated only at 8 weeks (Fig. 6b). The downregulation of these specific lipid mediators in transgenic animals was not due to a reduced expression of the genes encoding for their enzymes (15-LOX and 5- LOX respectively), named *Alox15A* and *Alox15B*, as shown by qRT-PCR (Supplementary Fig. 5b).

## Discussion

Our study demonstrates that epithelial GPR120, mainly expressed by goblet cells, plays an essential role in maintaining mucosal barrier integrity by regulating mucus production, and in preventing the inappropriate bacteria recognition and penetration that are linked to cancer development^29^. In this context, the inner mucus layer plays a crucial role in defending host against pathogens^30^. However, we are still only at the beginning of understanding the mechanism by which the integrity of the inner mucus layer is maintained by the host, particularly when challenged with microorganism penetration.

Gut epithelial cells are efficiently protected by the mucus, mainly consisting of mucin 2 (Muc2)^31^. The mucus is composed of two distinct layers: an inner mucus layer firmly attached to the epithelium, and an outer non-attached layer, easy to remove^32,33^, and besides mucins, it is characterized by other proteins that help to maintain the epithelial barrier integrity^33^. Our proteomic analysis performed on the mucus of healthy GPR120^∆IEC^ mice and WT littermates revealed interesting differences in the mucus composition of the two groups. Among the proteins found expressed in both groups, we observed Muc2 to be significantly downregulated in healthy GPR120^∆IEC^ than WT littermates. This is important, because downregulation of Muc2 expression was associated with early carcinogenesis events in colon cancer^34,35^, which is consistent with the fact that at later stages of CRC development no differences in colonic *Muc2* levels were observed between tumor bearing GPR120^∆IEC^ and WT mice.

Among proteins that were exclusively present in the mucus of GPR120^∆IEC^ mice, worthy of attention are Myosin 11 (Myh11), Myosin 9 (Myh9), and Vinculin (Vcl). In fact, myosins are essential regulators of cellular homeostasis and tissue integrity, playing multiple roles in cell polarity, division, motility, and mechanotransduction; some of them have been shown to play important roles in establishing normal intestinal barrier, and protection from mucosal inflammation in vivo^36^*.* A study from Wallace and colleagues showed that Myh11 mutation disrupts epithelial architecture in the developing zebrafish intestine, interfering with cells integrity and inducing epithelial cells to acquire an invasive phenotype^37^. Furthermore, Myh6 and vinculin are part of a molecular apparatus responsible for generating the cohesive cell–cell contacts that distinguish epithelial biogenesis in vitro^38^*.* On the other hand, Vinculin is reported to be involved in pathogenic bacteria invasion, a phenomenon well characterized for Gram-negative *Shigella*, but less known for other bacterial species^39^. These proteins, together with the up-regulation of some SCFAs, could explain why epithelial deletion of GPR120 did not affect intestinal inflammation. Small Chain Fatty Acids produced as microbial metabolites, namely acetate, propionate, and butyrate, can modulate many biological responses like inflammation, through two mechanisms; while one is associated with the inhibition of nuclear class I histone deacetylases, the other acts through the direct activation of some G-protein coupled receptors^40^. Among SCFAs, butyric acid is the only one stands out as upregulated in transgenic mice, and it is extensively studied for its controversial effect named as “butyrate paradox”, in which a wealth of experimental evidence has demonstrated the inhibitory effect of butyrate on tumorigenesis^41^ and the capacity to mediate anti-inflammatory effects^42^, but this SCFA can also paradoxically stimulate mucosal proliferation under certain conditions^43^.

In parallel, we observed changes in the intestinal microbiota of healthy and AOM/DSS-induced GPR120^ΔIEC^ versus WT animals, whose alteration may either be the cause or the consequence of the altered mucus composition. To date, there is no consensus on the composition of the gut microbiota in preneoplastic lesions or CRC. However, some bacterial species such as *Bacteroides fragilis*, and *Escherichia coli*, have been correlated with CRC carcinogenesis^44,45^. Among the bacteria significantly more abundant in healthy GPR120^ΔIEC^ versus WT animals, we found an OTU belonging to the genus *Bacteroides*. Members of this bacterial taxon have been recognized among the “drivers” of CRC, and their metabolites can damage the DNA of colonic epithelium, leading to initiation of tumor development^44^, which is consistent with the increased susceptibility of GPR120^ΔIEC^ mice to the AOM/DSS protocol. Additionally, we found a large decrease in *Firmicutes* and *Actinobacteria* with concomitant relative expansion of *Proteobacteria,* as reported previously in CRC patients^46^. Furthermore, a higher abundance of *Akkermansia muciniphila* was noticed in healthy GPR120^ΔIEC^ compared to WT animals. *A. muciniphila*, a Gram-negative and anaerobic bacterium of the intestinal mucus layer^47^, besides to degrade host mucin into various products (e.g., short chain fatty acids), regulates host biological functions, such as glucose and lipid metabolism, the expression of mucus-related genes^47^, and maintaining gut barrier function^48^, that are key processes in the regulation of intestinal homeostasis. Therefore, the lack of GPR120 favors CRC development by conditioning the microbial composition and altering epithelial barrier function. However, the mechanisms by which the loss of GPR120 leads to a dysregulated barrier function need to be better clarified. It is plausible that, in addition to mucus production, GPR120 regulates the attachment of epithelial cells to the underlying basal lamina maintaining cell polarity and helping Cell-Extra Cellular Matrix (ECM) adhesion. Indeed, in RNA-sequencing performed on primary epithelial cells of healthy transgenic mice, no modulation in genes encoding for proteins involved in cell–cell interaction was observed, evincing a downregulation of genes encoding for ECM proteins. This could further explain the reduction of TEER observed in GPR120-silenced cells, in which the dysregulation of cell-basement membrane interactions may make the barrier function moots.

To date, the role of GPR120 receptor in human CRC remains still unclear. Wu and colleagues found that the expression of *FFAR4,* the gene encoding for GPR120, is upregulated in CRC tissue compared to adjacent non-cancerous areas^14^, and that the expression of the receptor increases as the clinical stage of advanced cancer. In sharp contrast, Zhang and colleagues demonstrated that GPR120 suppresses cell proliferation and promotes apoptosis in CRC cells treated with ω-3 PUFA^49^. Our findings demonstrate that *FFAR4* is significantly downregulated in human CRC by using the GENT2 database, but also that the expression of the receptor decreases increasing the grade of lesion as invasive cancer. Accordingly, conditional deletion of GPR120 in the intestinal epithelium significantly increased the density of small-sized proliferative lesions, but not promoted the development of malignant ones. Nevertheless, we cannot exclude that the absence of adenocarcinomas could due to short observation window of the experiment. The significantly higher number of proliferating cells in colon from AOM/DSS-induced transgenic mice, and the proliferation assay in vitro upon GPR120 siRNA silencing, strongly indicated that epithelial loss of epithelial GPR120 promotes CRC growth and not differentiation. Indeed, no difference in the number of low- and high-grade adenomas has been found between GPR120^ΔIEC^ and WT mice in according to previous studies^50–52^. Wnt signaling pathway, whose dysregulation may lead to tumor development and growth^53^, was found altered in absence of GPR120. Biological processes related to the Wnt pathway have been indeed found enriched in the epithelium of healthy GPR120^ΔIEC^ versus WT mice. In addition, *CTNNB1* gene encoding for β-catenin was significantly up-regulated in siRNA treated cells with most of its expression localized in the cytoplasm of intestinal epithelial cells. Most likely, β-catenin accumulation results from the absence of the receptor in the intestinal epithelium that allows for hyperproliferation. Interestingly, GPR120 it is also expressed in progenitor cells, in which two major molecular events occur: the expression of the gatekeeper tumor suppressor gene adenomatous polyposis coli (APC) and the simultaneous silencing of the Wnt/ β-catenin signaling^54^. This, could open another investigation frame about how GPR120 and the Wnt signaling may be interconnected. However, a deeper investigation about the role of GPR120 in cancer progression is needed.

Worthy of interest is our evidences showing that epithelial GPR120 is important to slow down the cancer progression. In this context the contribution of ω-3 PUFAs may make the difference. Many clinical studies have confirmed the inverse correlation between CRC and ω-3 PUFAs intake^55–57^. Consistently with that, our results showed a down-regulation of ω-3 PUFAs, and it is even in line with evidences in which high ratio ω-3-derived lipids may be considered as biomarker promoting for metastatic CRC^58^. The previous studies showed that CRC cells preferentially uptake ω-3 PUFAs^59^, and that a correlation between the expression receptors and its ligands^60^ is possible. Taken together with our findings, in which the absence of the receptor is associated with ligands’ downregulation, we strongly believe in a possible autocrine loop, with consequent reduction of these lipids the uptake is not possible and then cancerous cells cannot progress as invasive cancer. Finally, the ω-3 PUFAs have even effects on intestinal permeability: EPA and DHA have been shown to improve barrier integrity in in vitro studies^61^. Moreover, many studies support the role of EPA and DHA as modifier of proliferative pattern in epithelial cells in patients with sporadic adenomatous polyps, but the mechanism exerting this effect in colonic mucosa is not clear yet^62^.

In conclusion, in the present study we provided for the first-time evidence of the epithelial GPR120 role in maintaining the protective inner mucus layer. Its loss promotes epithelial barrier impairment, dysbiosis and bacterial translocation, and hyperproliferation of epithelial cells, but on the other hand prevents tumor progression. Although, further studies are needed to elucidate better the molecular involvement of GPR120, our data pave the way to future applications of GPR120 also as a useful marker in clinics.

## Materials and methods

### Animal studies

In order to generate mice with a conditional deletion of GPR120 in the intestinal epithelium, *Ffar4*^flox/flox^ mice (in which Exon 1 and approx. 1.5 kb of sequence upstream of exon 1 (promoter region) have been flanked by loxP sites) were crossed with *Villin-cre* transgenic mice. In the following steps, *VillinCre*-*Ffar4*^flox/+^ were crossed with *VillinCre*-*Ffar4*^flox/+^ to obtain *VillinCre*-*Ffar4*^flox/flox^ mice (named GPR120^ΔIEC^) and *VillinCre*-*Ffar4*^+/+^ (named WT) littermates, that were used for the experiments. In these mice, deletion of exon 1 and proximal promoter results in loss of function of the *Ffar4* gene, by preventing transcription of the *Ffar4* mRNA and by deleting the GPR120 transmembrane domains 1 to 4. The mice were generated by ordering ES cells Ffar4^tm2a(EUCOMM)Hmgu^ (KO first allele (reporter-tagged insertion with conditional potential) from https://www.mousephenotype.org/data/genes/MGI:2147577 website. Then, the ES cells were injected in blastocysts and implanted in mice in order to obtain the chimera. To evaluate if Cre excision occurred between the external loxP sites, we performed PCR analysis. Then, depending on the product size we were able to understand if the chimera carried the germline mutation. The scheme of modified allele and the information about the product size are reported in Supplementary Material.

All mice were maintained in a specific pathogen free (SPF) facility certified by Charles River Laboratories International. Housing was temperature controlled, with a 12 light/12 dark hour cycle. Procedures involving mice conformed to institutional guidelines in agreement with national and international law and obtained ethical approval from the Italian Ministry of Health. The study follows the ARRIVE guidelines for the in vivo studies carried out on animals.

### Genotyping of mouse tail DNA by PCR

Pups were tailed and toed (for identification) around 3-weeks of age. Transgenic animals (GPR120^ΔIEC^) were distinguished from heterozygous *VillinCre*-GPR120^+/−^ (Het) and wild-type *VillinCre*-GPR120^+/+^ (WT) littermates by PCR (Supplementary Fig. S6). The DNA was collected from the tail tissue by lysing the samples with 200 µL of Sodium Hydroxide (NaOH, 60 mM), heating at 98 °C for 20 min, then adding 60 µL of neutralization solution containing TRIS Hydrochloride (TRIS–HCl, 1 M, pH8). Primers designed to specifically amplify the sequence of interest (see table below), were added with GoTaq Polymerase (Promega, cat. n° M3001) and DNA to the master mix, according to manufacturer’s instructions. After the PCR reaction, products were separated on a 2% agarose gel and visualized with Bio-Rad ChemiDoc Imager.

**Table:** 

Gene	Forward primer	Reverse primer
Ffar4	5’CAAGTCAATCGCACCCACTT 3’	3’CGGCTTTGGTCAGATCCTTG 5’
VillinCre	5’CAAGCCTGGCTCGACGGCC 3’	3’ CGCGAACATCTTCAGGTTCT 5’

### AOM/DSS-induced colitis-associated colorectal cancer

CAC was induced by a single intraperitoneal injection of azoxymethane (AOM, 10 mg/kg, Sigma-Aldrich) in 7 weeks-old C57BL/6 mice and kept on regular water for 7 days. After 7 days, mice were subjected to four oral cycles of 2% of dextran sulfate sodium (DSS) (molecular mass, 40 kDa; MpBio, cat. n°160110) in drinking water for one week followed by one week of normal drinking water. Body weight, stool consistency and rectal bleeding were monitored every two days. The consistency of stool was scored as 0-normal, 1-soft formed, 2-diarrhea-like, 3-watery, 4-not formed and with blood. Presence of blood was detected using Hemoccult kit (Beckman Coulter, cat. n°395034) and scored with increasing numbers starting from 0-no blood to 4-copious and eye-visible amount of blood. Mice were sacrificed after 57 days from the first DSS exposure by CO_2_, after endoscopy. Colitis severity was scored using a disease activity index (DAI) score based on daily evaluation of body weight, diarrhea and presence of blood in stools. Scoring of tumor development was evaluated by a high-resolution video miniendoscope (Karl Storz, Tuttlingen, Germany), on the basis of the tumor size and the number of tumors, as described previously^63^. At sacrifice, part of the colon was flash frozen (at − 80 °C) for mRNA analysis and part was fixed in formalin overnight, embedded in paraffin and sectioned for histological analysis.

### Immunohistochemistry and Immunofluorescence stainings

For immunohistochemistry, three-micrometer formalin-fixed paraffin-embedded colon sections were cut, dewaxed and hydrated. Antigen retrieval was conducted using Citrate buffer (pH6, 10 mM) in warm bath at 98 °C for 20 min. Endogenous peroxidase was blocked for 15 min at room temperature, and then non-specific sites were blocked by Rodent Block (BioCare Medical, cat. n° RBM961G) or Background Sniper (BioCare Medical, cat. n° BS966). Slides were next incubated with the primary antibody against Ki67 [rabbit anti-human/mouse (Abcam cat n° ab15580) 1:600 dilution] or ß-catenin [rabbit anti-human/mouse (Abcam cat n°ab6302) 1:800 dilution] or GPR120 [rabbit anti-human/mouse (Abcam cat n° ab223512) 1:300 dilution] for two hours at room temperature. Subsequently, sections were incubated with the secondary antibody (MACH1 universal HRP-polymer detection, cat. n° M1U539G) at room temperature for 30 min, followed by (Phosphate Buffered Saline –Tween 25 mM) PBS-T washing. The immunostaining was visualized with brown DAB (Dako, Carpinteria, CA, USA) and counterstained with Hematoxylin, dehydrated and covered with coverslips.

For immunofluorescence staining, frozen sections were fixed with Paraformaldehyde (PFA) 4% and then permealized using PBS + 0,01% Triton. After incubation with primary anti-GPR120 (1:200 dilution), anti-Jam A^24,64^(1:20 dilution) anti-Pan Cytokeratin [(mouse anti-human, Abcam cat n° ab86734) 1:100 dilution] and secondary antibodies (anti-rabbit Alexa Fluor 647-conjugated antibody and anti-mouse Alexa Fluor 488-conjugated antibody respectively, 1:500 dilution, Life Technologies), slides were counterstained with DAPI. Control slides were obtained by omitting the use of primary antibodies. Images were taken with Leica SP8I Confocal Microscopy.

### Histological analysis

Images were acquired using the VS120 DotSlide system (Olympus), and immunostainings were evaluated by ImageJ (version 2.3.0/1.53f.; www.imagej.net/contributor), using both Olympus and IHC plugins. Olympus plugin allowed to open the file without affecting the resolution of the image; the IHC protocol of ImageJ software automatically detected DAB positive areas; upon conversion of the picture into 8-bit, the brown color was adjusted with threshold and then positive particles were analyzed. For histological analyses, FFPE sections were stained with Hematoxylin and Eosin, according to the standard procedure. The stained sections were scored by an experienced pathologist in a blinded manner.

### In vivo quantification of intestinal permeability

Intestinal permeability was evaluated by perfusion of the mouse intestine with the azo Evans Blue dye, as previously described^65^. 8 weeks old mice were anesthetized with a mixture of ketamine and xylazine. The abdominal quadrant of peritoneum was opened in correspondence of the caecum, which together with the proximal region of the colon was completely exposed. A tiny cut in the proximal region of the colon nearby the caecum was performed and a catheter inserted into the lumen and into the anus. The luminal contents were washed out gently with 4–5 mL of warm PBS. Afterwards, the colonic lumen was perfused with Evans blue solution 0.1% into flowing through the two catheters 1 ml/min for five minutes and then washed to remove the excess of Evans blue dye. After this time, mice were sacrificed by decapitation and the colon was rapidly dissected out, opened, rinsed with PBS, dried on filter paper at 37 °C for 24 h, and then weighed and incubated with 3 mL of Dimethyl Formamide at 50 °C for 24 h. The amount of the Evans blue into the solution was quantified as the spectrophotometric optical density (OD) at 620 nm wavelength and the colon permeability evaluated as the ratio between optical density and the weight of the colon.

### Fluorescence in situ hybridization

Healthy GPR120^ΔIEC^ and WT 8 weeks old mice were fed for two weeks with cornmeal mush, then sacrificed by CO_2_ and colon tissues embedded in OCT without any washing steps. Cryosections of 8 μm were fixed with Metacarnoy solution (75% Methanol + 25% Acetic Acid) for 4 h at room temperature, then washed in Ethanol (EtOH) and dried in the oven for 30 min. The hybridization area was marked with diamond cutter in order to localize it during the rest of procedure. The fluorescence in situ hybridization (FISH) assay was performed using an Alexa Fluor 555 (Invitrogen)–conjugated eubacterial probe (EU 622773). Each slide was stained with the bacterial probe diluted in freshly prepared Hybridization Buffer [Sodium Cloride (NaCl) 2 M; TRIS HCl 1 M; Sodium Dodecyl Sulfate (SDS) 10%; H_2_0). The hybridization chamber was prepared and then loaded into DAKO machine overnight. Rubber cement was removed to wash slides in water-bath at 55 °C for 20 min. Washes in hybridization buffer + 2%BSA were performed to remove nonspecific hybrids. Colon sections were incubated with primary [Muc2 (Santa Cruz, B306.1; cat n° sc-59859), mouse anti-human/mouse, 1:25 dilution] and secondary (goat anti-mouse, 1:1000 dilution, Life Technologies) antibodies at room temperature for 1 h and then stained with DAPI. Images were acquired with an Olympus FV1000/TIRF microscope.

### In vitro measurement of epithelial barrier integrity

Transepithelial/transendothelial electrical resistance (TEER) is a quantitative technique that allows to evaluate the epithelial barrier integrity in cell culture models. The setup consists of a cellular monolayer cultured on semipermeable filter which defines the apical portion from the basolateral one. It is composed by two electrodes: one is placed in the upper compartment and the other in the lower, separated by the cellular monolayer. The ohmic resistance determined by applying a direct current (DC) voltage to the electrodes gives an estimation of the cellular monolayer permeability. The higher the resistance, the lower the permeability^66^. This system has been used on monolayers of Caco-2 and LoVo cells, in which cells of our interest were plated in the lower compartment at ~ 60% of confluency, treated with siRNA and, 48 h post-transfection, the measurement was performed.

### Collection of mucus layer, protein gels and liquid chromatography in tandem mass spectrometry (LC–MS/MS)

Healthy GPR120^ΔIEC^ and WT 8 weeks old mice were sacrificed and colon was gently washed with PBS. The colon was opened longitudinally and mucus was scraped off the epithelial layer and collected by pipette. Collected mucus were mixed with protease inhibitors (PI 50x; PMSF 100x; NaVPO 200x) and then stored at − 20 °C until use. Two-dimensional electrophoresis procedure is schematized in Supplementary Fig. S7.

2-DE: About 250 µg of extracted proteins were dissolved in 125µL of rehydration buffer [Urea 8 M, 4% CHAPS (w/v), Dithioerythitol (DTE) 65 mM, 0,8% carrier ampholytes (v/v), 0,5% bromophenol blue] and loaded onto 7 cm IPG strips, with nonlinear (NL) pH 3–10 or linear pH 4–7 gradient range (GE Healthcare, UK). Strips were rehydrated without applying voltage for 1 h at 20 °C. The first dimensional IEF was carried out at 15 °C using an Ettan IPGphor 3 system (GE Healthcare, UK). Three hours later, the IEF run was stopped to allow positioning of small pieces of paper between the IPG strip and electrodes. Reduction and alkylation steps were performed between the first and the second dimension. The focused IPG strips were incubated for 15 min at room temperature in Urea 6 M, 2% (w/v) SDS, Tris 50 mM pH 6.8, glycerol 30%, containing 2% (w/v) DTE, followed by a second incubation of 15 min in the same buffer containing 2,5% (w/v) iodoacetamide and 0,5% bromophenol blue. At the end of the IEF step, strips were hold in place with 0,4% low melting temperature agarose and loaded onto 8 × 6 cm slabs, 12,5% SDS polyacrylamide gels. Electrophoresis was carried out at a constant current of 10 mA per gel in a PROTEAN II xi 2-D BioRad Cell equipment (Berkeley, California), until the buffer front line was 1 mm from the bottom of the gel.

#### LC–MS/MS

Identification of the protein(s) under the spots was carried out on an LC–MS/MS (Thermo Finnigan, USA) system consisting of a thermostated column oven Surveyor autosampler controlled at 25 °C, a quaternary gradient surveyor MS pump equipped with a diode array detector, and a Linear Trap Quadrupole (LTQ) mass spectrometer with electrospray ionization ion source controlled by Xcalibur software 1.4 (Thermo Fisher Scientific). Analytes were separated by RP-HPLC on a Jupiter (Phenomenex, USA) C18 column (150 × 2 mm, 4 µm, 9 Å particle size) using a linear gradient (2–16% solvent B in 60 min) in which solvent A consisted of 0.1% aqueous formic acid (FA) and solvent B consisted of Acetonitrile (ACN) containing 0.1% FA. Flow-rate was 0,2 mL/min. Mass spectra were generated in positive ion mode under constant instrumental conditions: source voltage 5.0 kV, capillary voltage 46 V, sheath gas flow 40 (arbitrary units), auxiliary gas flow 10 (arbitrary units), sweep gas flow 1 (arbitrary units), capillary temperature 200 °C, tube lens voltage − 105 V. MS/MS spectra, obtained by CID studies in the linear ion trap, were performed with an isolation width of 3 Th m/z, the activation amplitude was 35% of ejection RF amplitude that corresponds to 1.58 V. Data processing was performed using Peaks studio software^67^ (Thermo Fisher Scientific; https://www.thermofisher.com/order/catalog/product/OPTON-30965#/OPTON-30965).

### Western blot

Samples obtained from the two-dimensional electrophoresis protocol were lysed in RIPA buffer, followed by protein quantification. Upon separation on electrophoresis gel, proteins were transferred onto a nitrocellulose membrane by using a Trans-Blot Turbo Transfer System (BioRad). B-actin was used as loading control. Nonspecific binding was blocked with Tris-buffered saline (TBS) containing 1% non-fat dried milk for 1 h, followed by overnight incubation at 4 °C with the mouse anti-human MUC2 antibody, that recognizes also the mouse protein. Membranes were washed and then incubated for 2 h at room temperature with the appropriate HRP-conjugated secondary antibody (1:3000; GE Healthcare). Membranes were then incubated with Immobilon Western Chemilum (Millipore) and bands were detected by Chemidoc (Bio-Rad Laboratories), using Quantity One software (Bio-Rad Laboratories; https://www.bio-rad.com/it-it/product/quantity-one-1-d-analysis-software?ID=1de9eb3a-1eb5-4edb-82d2-68b91bf360fb).

### Microbiome analysis

Stool samples were collected from healthy mice and immediately frozen at − 80 °C. Metagenomic DNA was extracted from about 50 mg of feces using PowerSoil DNA isolation Kit (MO Bio Laboratories) according to manufacturer’s instructions. Subsequently, the bacterial community structure was profiled by 16S rRNA gene profiling. In brief, Pro-bio_Uni and Probio_Rev primers were used to amplify a partial region of the 16S rRNA encompassing the V3 variable region, then amplicons were sequenced using Illumina MiSeq System^68^.

### Analysis of short chain fatty acids and lipid mediators

Stool samples were collected at three different time points: time 0 = 0 weeks from AOM injection (healthy, n = 10), time 4 = 4 weeks from injection when tumor starts to grow (n = 4) and time 8 = 8 weeks after injection (end of experiment, n = 4) and immediately frozen at − 80 °C until use. SCFAs were quantified following the protocol published by Hoving et al^69^. Briefly, aqueous feces extract was prepared, a mix of isotopically labelled internal standards (containing acetic acid-d4, propionic acid-d6 and butyric acid-d8) was added and analytes were derivatized using Pentafluorobenzyl Bromide. The analytes of interest were extracted in *n*-hexane and measured by GC–MS. Analytes were chemically ionized (CI) and the MS was operated in negative mode using a single ion monitoring (SIM) method.

From the same samples, lipid mediators were analyzed. Briefly, 20 µL aqueous feces extract was mixed with 60 µL MetOH and 3.2 µL of a mix of isotopically labelled internal standards (containing 50 ng/mL Prostaglandin E_2_-d4, Leukotriene B_4_-d4 and 15-HETE-d8 and 500 ng/mL DHA-d5) in MetOH. The sample was mixed, incubated at -20 °C for 20 min. and centrifuged (16.1 krcf, 10 min., 4 °C). 50 µL supernatant was diluted with 50 µL water, vortexed and measured by LC- MS/MS as described elsewhere^70^.

### In vitro cell proliferation, and siRNA transfection

Human colorectal cancer cell lines [Caco-2 (ATCC HTB-37), and LoVo (ATCC CCL-229)] were maintained in culture with DMEM medium (Merck, cat no SLM-241-B) containing 10% (v/v) Fetal Bovine Serum (FBS) and 1% antibiotics (Penicillin–Streptomycin), at 37 °C and 5% CO_2_.

For proliferation assays, cells were seeded in 6-well plates at 100.000 cells/well (~ 40% confluency), in complete medium and transfected with siRNA against the free fatty acid receptor 4 (*FFAR4*) or control scramble siRNA, by using Lipofectamine 2000 (Invitrogen, cat. n° 11668019), following manufacturer’s instructions. siRNA oligos and their scramble siRNA control were designed by using the siRNA Wizard Software 3.1 and are reported in Table 1.

**Table 1:** The siRNAs sequences.

Sample	Sequence
siRNA #1	5' GAGTGGCGTAAGCCGACTATT 3'
siRNA #2	5' GTGGCGTAAGCCGACTATTGA 3'
siRNA #3	5' GGCCTGGAGATGCACATTGTT 3'
Scramble	5' GAAGGACGCTGACGTTGATCT 3

Briefly, 2500 ng/μl of siRNA oligomers was diluted in 50 μl Gibco OptiMEM I Reduced Serum DMEM medium and 1 μl of Lipofectamine 2000 were diluted in the same amount of Gibco OptiMEM I Reduced Serum (amounts and volumes are given on a per well basis). After 5-min incubation, the diluted oligomers and the diluted Lipofectamine 2000 were combined and incubated for 20 min at room temperature. The oligomer-Lipofectamine 2000 complexes were then added to each well of 6-wells cell culture plates and incubated for 48 h at 37 °C in CO_2_ incubator. After two days, cells were trypsinized by Trypsin- EDTA and counted using a Burker chamber. Cells from each experimental group were used to extract total RNA, as described above.

### qRT-PCR

Total RNA was extracted from colon tissues and cancer cell lines using RNeasy Mini Kit (Qiagen, cat. no 74104), according to the manufacturer’s instructions. RT-PCR was performed using Fast SYBR Green Master Mix (Applied Biosystem, cat. n° 1408115) and Viia7 Detection system (Applied Biosystem). Glyceraldehyde 3-phosphate dehydrogenase (*GAPDH*) gene was used as internal control. Relative gene expression was determined by the 2^-ΔCt^ method. The specific oligonucleotide primers used are listed in Table 2.

**Table 2:** Primer sequences.

Gene name	Forward primer	Reverse primer
Ffar 4	5’ATTTTACAGATCACAAAGGCATC 3’	5’AGGCTTACCGTGAGCCTCTTC 3’
Alox 15a	5’GGCTCCAACAACGAGGTCTAC 3’	5’CCCAAGGTATTCTGACACATCC 3’
Alox 15b	5’ATGCAGGGTGAGAGTATCCAC 3’	5’TCCAGAGGTACTAAGGGGCTC 3’
Ctnnb1	5’ATGGAGCCGGACAGAAAAGC 3’	5’TGGGAGGTGTCAACATCTTCTT 3’
Muc 2	5’AGGGCTCGGAACTCCAGAAA 3’	5’CCAGGGAATCGGTAGACATCG 3’
Il1b	5’GAAATGCCACCTTTTGACAGTG 3’	5’TGGATGCTCTCATCAGGACAG 3’
Il6	5’CTGCAAGAGACTTCCATCCAG 3’	5’AGTGGTATAGACAGGTCTGTTGG 3’
Tnfa	5’CAGGCGGTGCCTATGTCTC 3’	5’CGATCACCCCGAAGTTCAGTAG 3’
Gapdh	5’AGGTCGGTGTGAACGGATTTG 3’	5’GGGGTCGTTGATGGCAACA 3’
FFAR4	5’AGACCTCGGAACACCTCCTG 3’	5’AGGCTTACCGTGAGCCTCTTC 3’
CCND1	5’GCTGCGAAGTGGAAACCATC 3’	5’CCTCCTTCTGCACACATTTGAA 3’
CTNNB1	5’CATCTACACAGTTTGATGCTGCT 3’	5’GCAGTTTTGTCAGTTCAGGGA 3’
GAPDH	5’GGAGCGAGATCCCTCCAAAAT 3’	5’GGCTGTTGTCATACTTCTCATGG 3’

### Cell cycle analysis and apoptosis by FACS

For cell cycle analysis, scramble siRNA-, and siRNA-transfected cells were washed with PBS and incubated in EtOH 80% for 30 min. After centrifugation and washing steps, cells were resuspended in DAPI/TX-100 solution (9 mL PBS; 1 mL Triton X-100; 10 μL DAPI 1 μg/mL) and incubated for 30 min at room temperature. Cell cycle analysis was next performed by FlowJo v.10.6.1 software, which provides a simple interface to perform sophisticated univariate DNA/Cell Cycle analysis using the Dean-Jett-Fox model^71^.

Quantification of apoptotic cells was performed using the Annexin V-FITC Kit (Abcam, cat n° ab14082) and 7-AAD (cat n° 559925), for the exclusion of nonviable cells and detectable in far red, following the manufacturer’s instructions. Briefly, 1 × 10^5^ cells were incubated with 5 µl of both Annexin V and 7-AAD in the proper buffer for 5 min in the dark and then acquired by FACS and analyzed by using FlowJo v.10.6.1.

### Isolation of epithelial cells from mouse colon

Colon tissues were collected from healthy wild-type and transgenic 8 weeks old mice and flushed with ice-cold PBS. Tissue was cut in small pieces and washed several times by inverting the 15-ml conical tube. After dissociation in 0,5 M EDTA for 30 min on an orbital shaker, the tube was shaken for 5 min by hand to dissociate epithelium from the basement membrane. The solution was filtered through a 100 μm filter to remove the villus fraction. After two centrifugations of 10 min at 150 g, 4 °C, the resulting pellet containing epithelial cells was stored at − 80 °C until use. Total RNA was extracted as described above and used for RNA-seq.

### Human samples

Frozen tissue biopsies of 9 normal tissues, 9 adenomas, and 17 primary adenocarcinomas were obtained from fresh tissue biobank collection at Humanitas Clinical and Research Center, and proceeded for the mRNA extraction. Healthy tissue was collected at a distance of at least 10 cm from tumor lesion. No patient had received any therapy before surgery.

### Bioinformatic analysis

RNA-seq raw data were analyzed by bioinformatician, who provided GSEA report. These data were analyzed using Panther, ReviGo and Reactome in order to understand the most differentially expressed biological processes, pathways and genes in GPR120^ΔIEC^ compared to WT mice. Significant up and down-regulated genes were selected depending on their NES (Normalized Enrichment Score). To analyze the differential gene expression of *FFAR4* in normal and cancer tissues, we took advantage of the GENT 2 database (http://gent2.appex.kr/gent2/http://gent2.appex.kr/gent2/). This database compares *FFAR4* gene expression in various tissues but also its expression across cancer subtypes [like AJJC staging based on the extent of the tumor (T), the extent of spread to the lymph nodes (N), and the presence of metastasis (M)]. For the proteomic analysis, differentially enriched proteins were identified DAVE score + 2/− 2. For the microbiome analysis, the resulting sequence reads were managed by means of the bioinformatic pipeline Quantitative Insights Into Microbial Ecology (QIIME) version 1.7.0^72^ with the GreenGenes database (version 13.5), which allowed clustering of sequences into operational taxonomic units (OTUs).

### Statistical analysis

All statistical analyses were performed using Prism GraphPad Prism 8.0 and STATA16 software. Data are presented as the median ± 95% CI (confidence interval). Student *t*-test was used to examine differences between groups with normal distribution, and its non-parametric counterpart (Mann Whitney test) was used if assumptions were not satisfied. A p.value < 0,05 was considered statistically significant. The differences in the microbiota composition between wild type and mutant mice were inferred at the OTU level using a Wald test following read count normalization with the DESeq2 negative binomial distribution method.

## Supplementary Information

Supplementary Information 1.

Supplementary Information 2.

Supplementary Information 3.

Supplementary Information 4.

Supplementary Information 5.

Supplementary Information 6.

Supplementary Information 7.
