# Supplementary material for: GPR120 prevents colorectal adenocarcinoma progression by sustaining the mucosal barrier integrity
Source: Sci Rep. 2022 Jan 10;12:381. doi: 10.1038/s41598-021-03787-7 (PMC8748819; doi:10.1038/s41598-021-03787-7)

Supplementary Figure 1

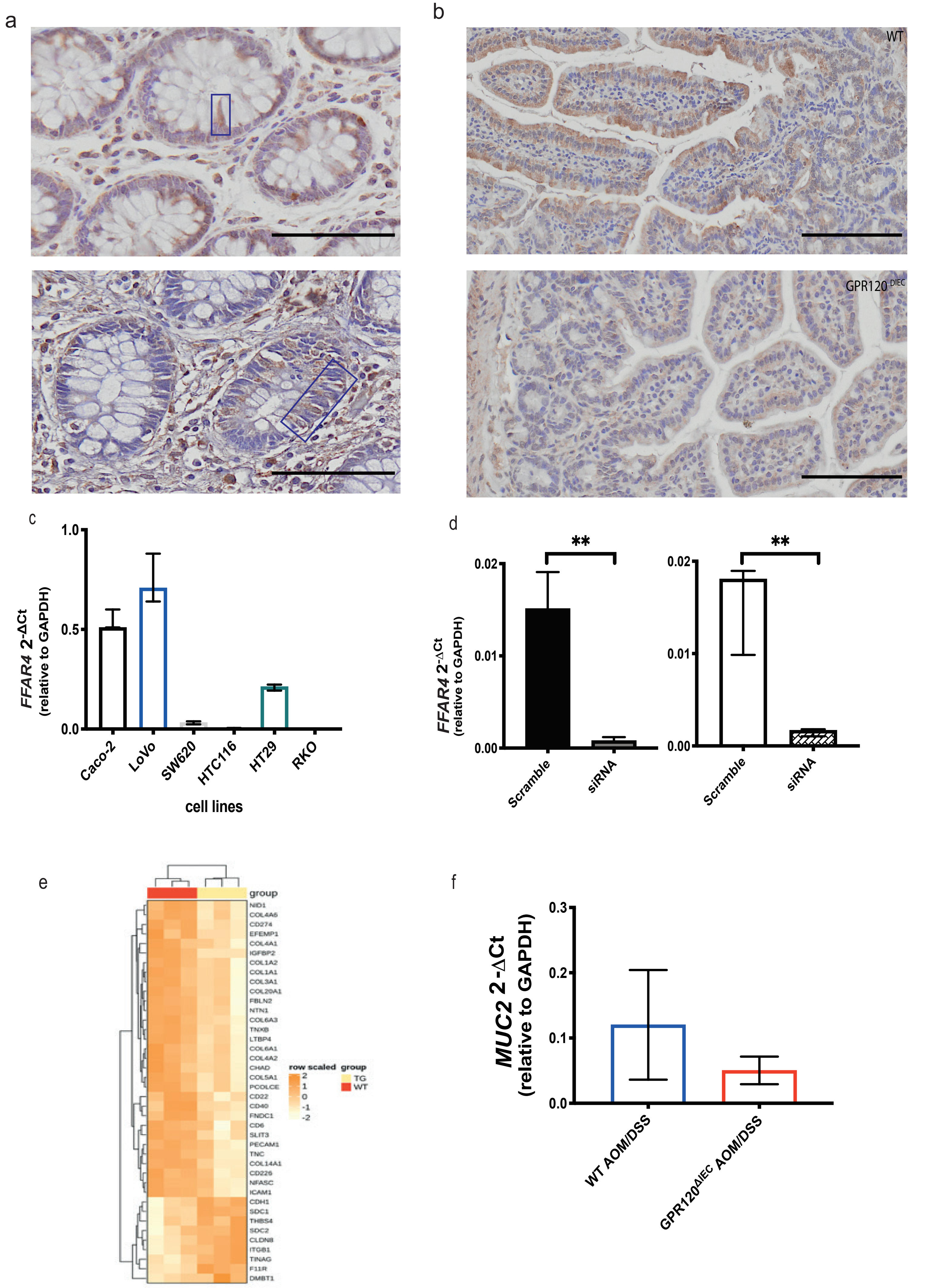

Supplementary Figure 2

a

OTU nr.	Taxonomy	T8 KO	T8 WT	log_2 Fold Change	padj
276629	*p_Bacteroidetes;c_Bacteroidia;o_Bacteroidales;f_S24-7;g_;s_*			-7.31	1.8E-03
187959	*p_Bacteroidetes;c_Bacteroidia;o_Bacteroidales;f_S24-7;g_;s_*			-6.13	4.6E-02
348038	*p_Bacteroidetes;c_Bacteroidia;o_Bacteroidales;f_S24-7;g_;s_*			-8.48	2.8E-04
416078	*p_Bacteroidetes;c_Bacteroidia;o_Bacteroidales;f_S24-7;g_;s_*			-10.69	3.5E-08
264534	*p_Bacteroidetes;c_Bacteroidia;o_Bacteroidales;f_S24-7;g_;s_*			-6.63	1.4E-02
273997	*p_Firmicutes;c_Erysipelotrichi;o_Erysipelotrichales;f_Erysipelotrichaceae;g_Allobaculum;s_*			-5.38	3.0E-02
268352	*p_Firmicutes;c_Erysipelotrichi;o_Erysipelotrichales;f_Erysipelotrichaceae;g_Allobaculum;s_*			-5.58	4.6E-02
272334	*p_Firmicutes;c_Erysipelotrichi;o_Erysipelotrichales;f_Erysipelotrichaceae;g_Allobaculum;s_*			-4.75	3.0E-02
581021	*p_Proteobacteria;c_Gammaproteobacteria;o_Enterobacteriales;f_Enterobacteriaceae;g_;s_*			7.80	4.6E-02

MIN MAX -11 +8

b

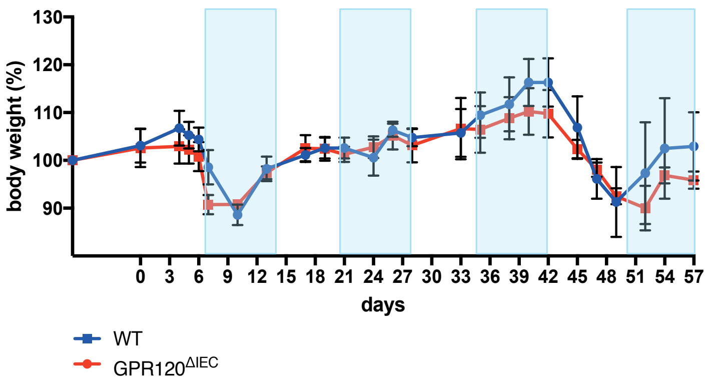

c

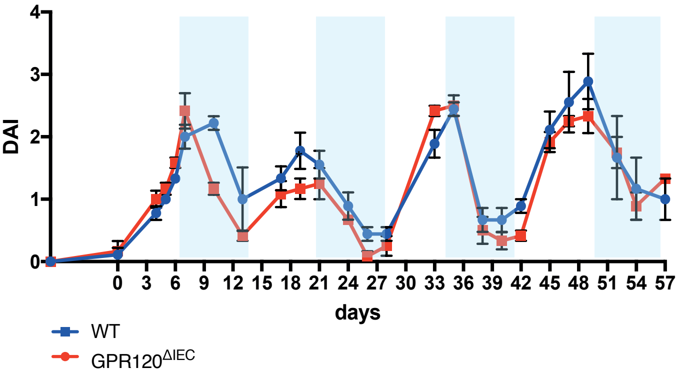

d

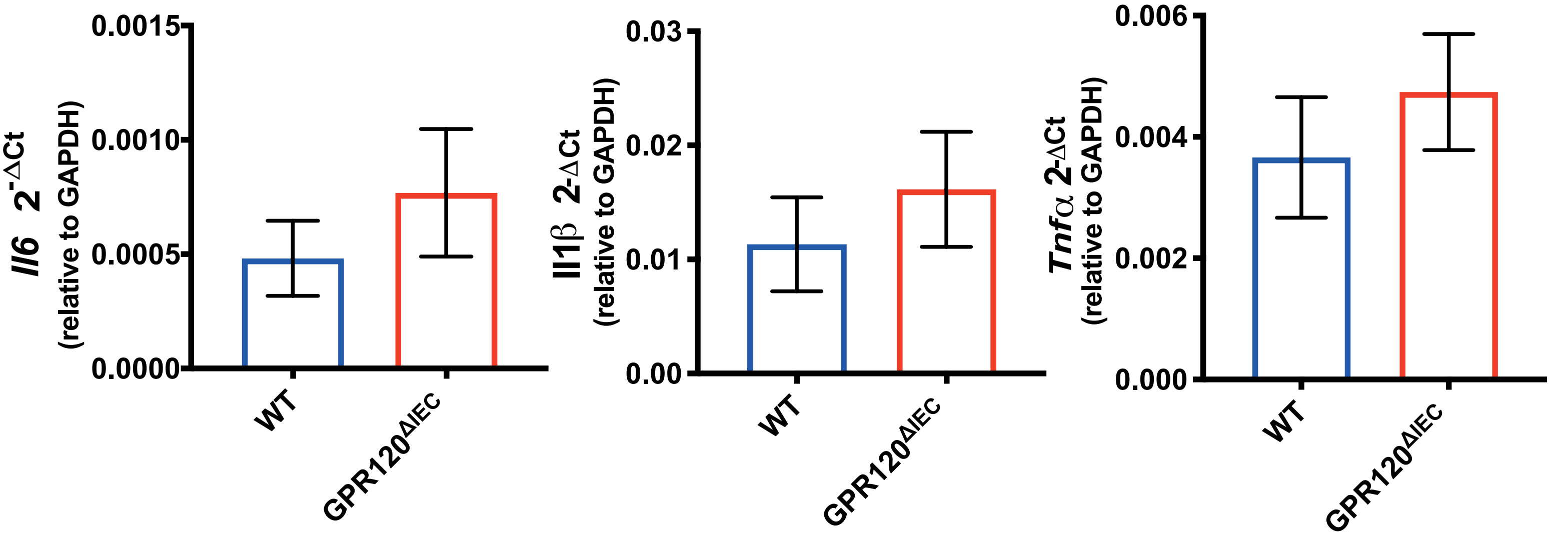

Supplementary Figure 3

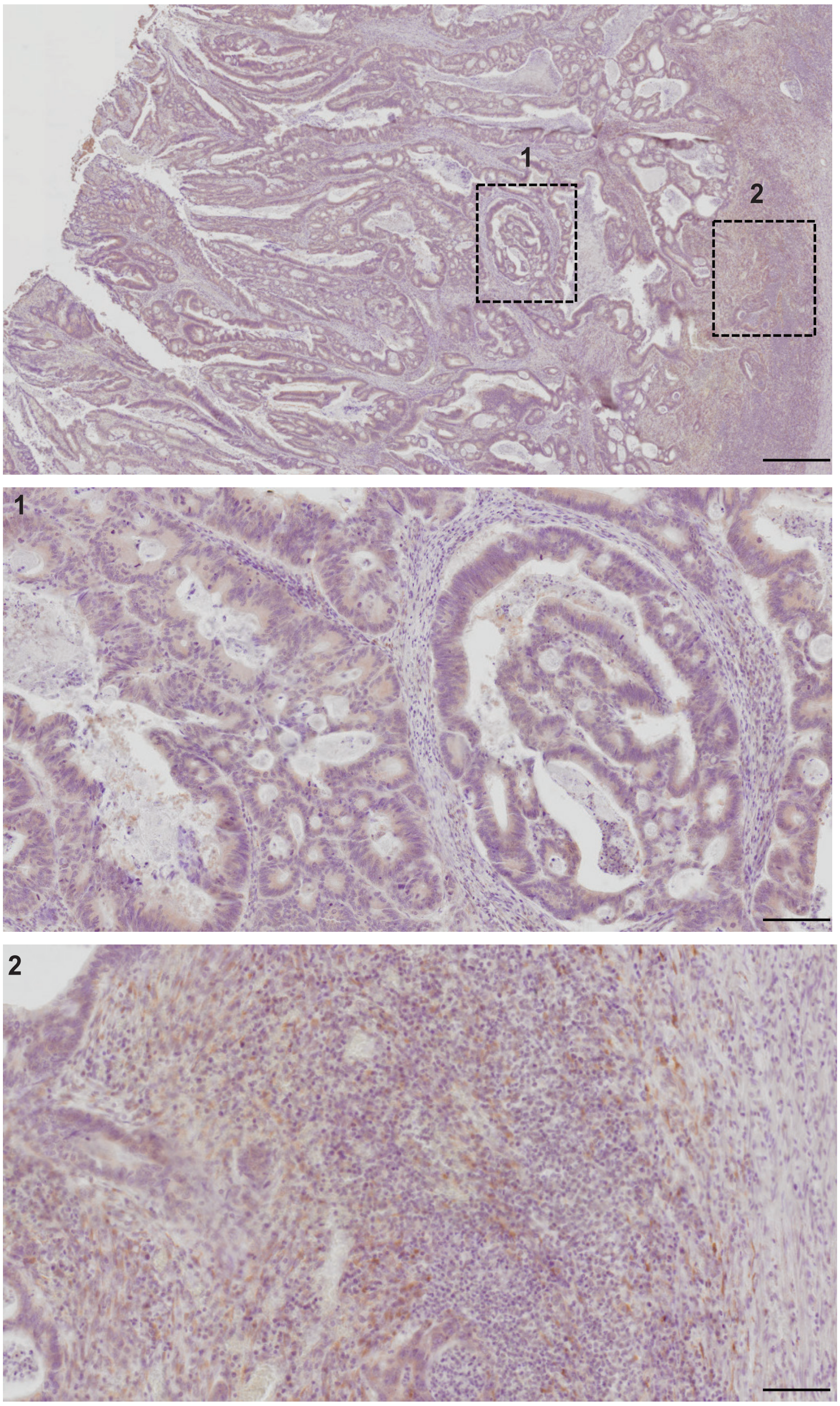

Supplementary Figure 4

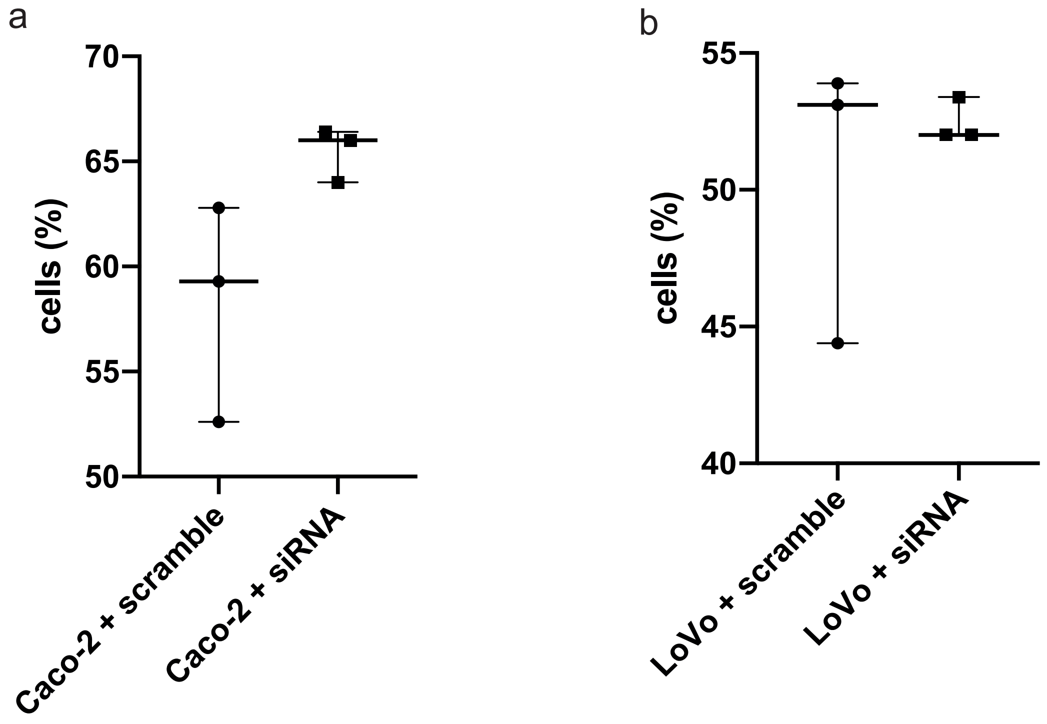

Supplementary Figure 5

a

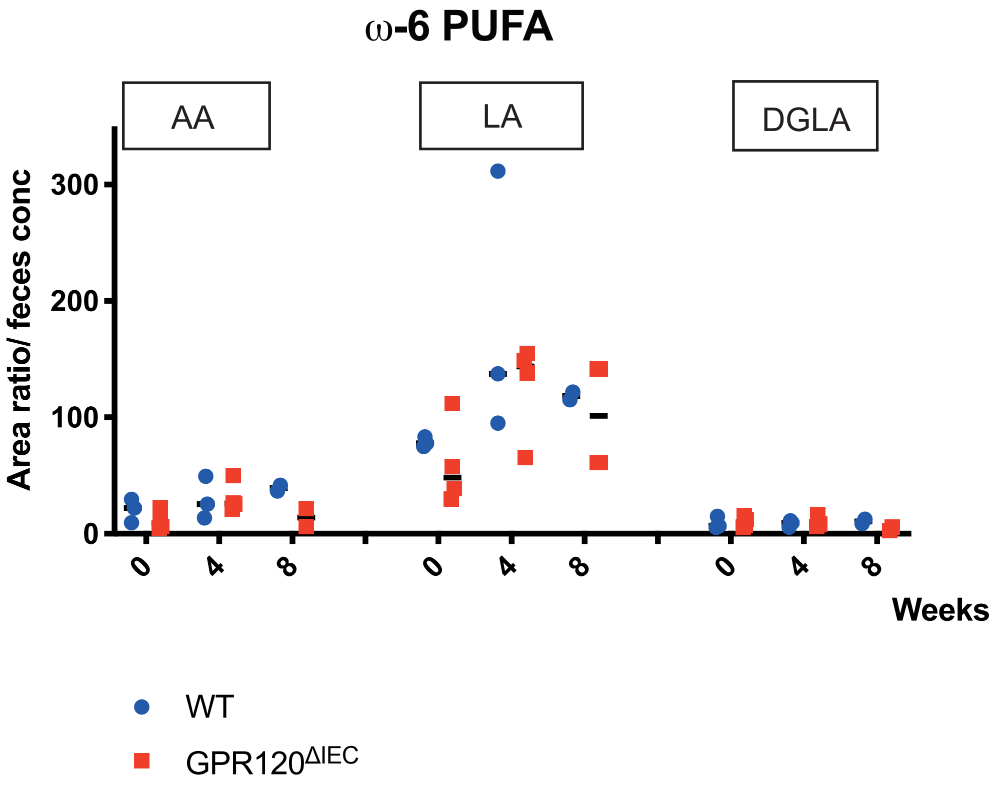

b

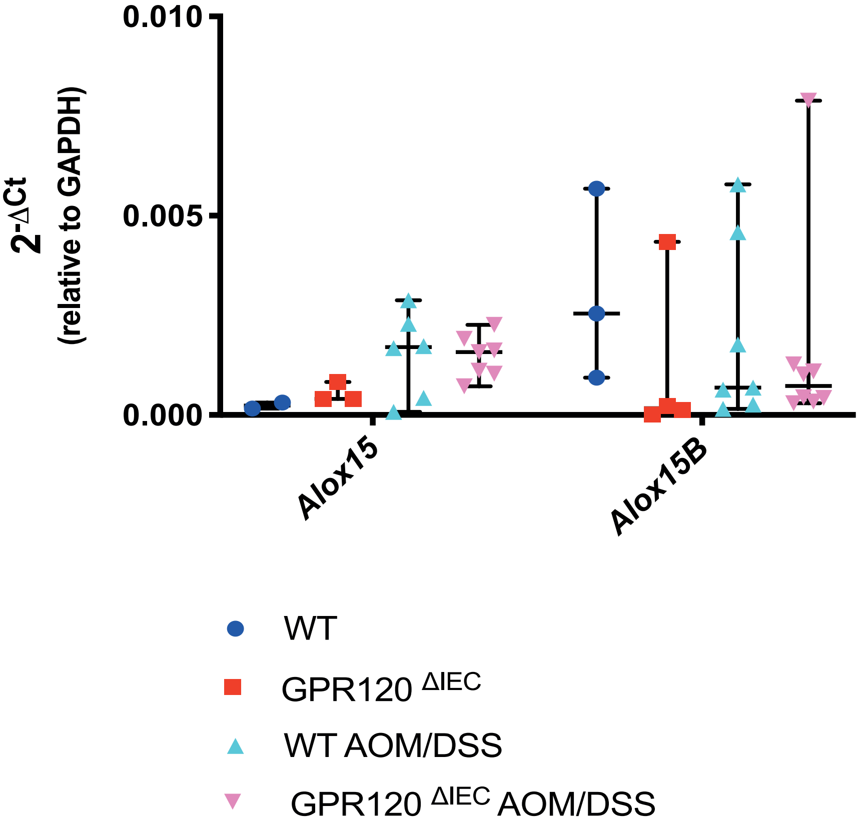

Supplementary Figure 6

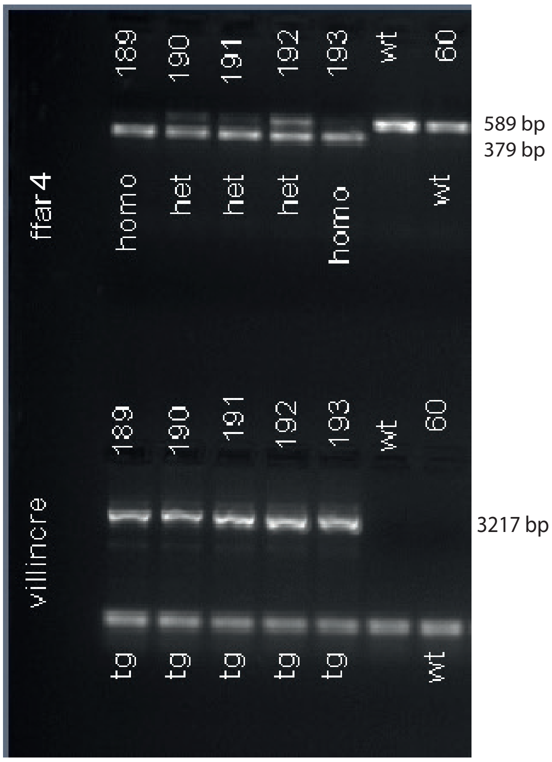

Supplementary Figure 7

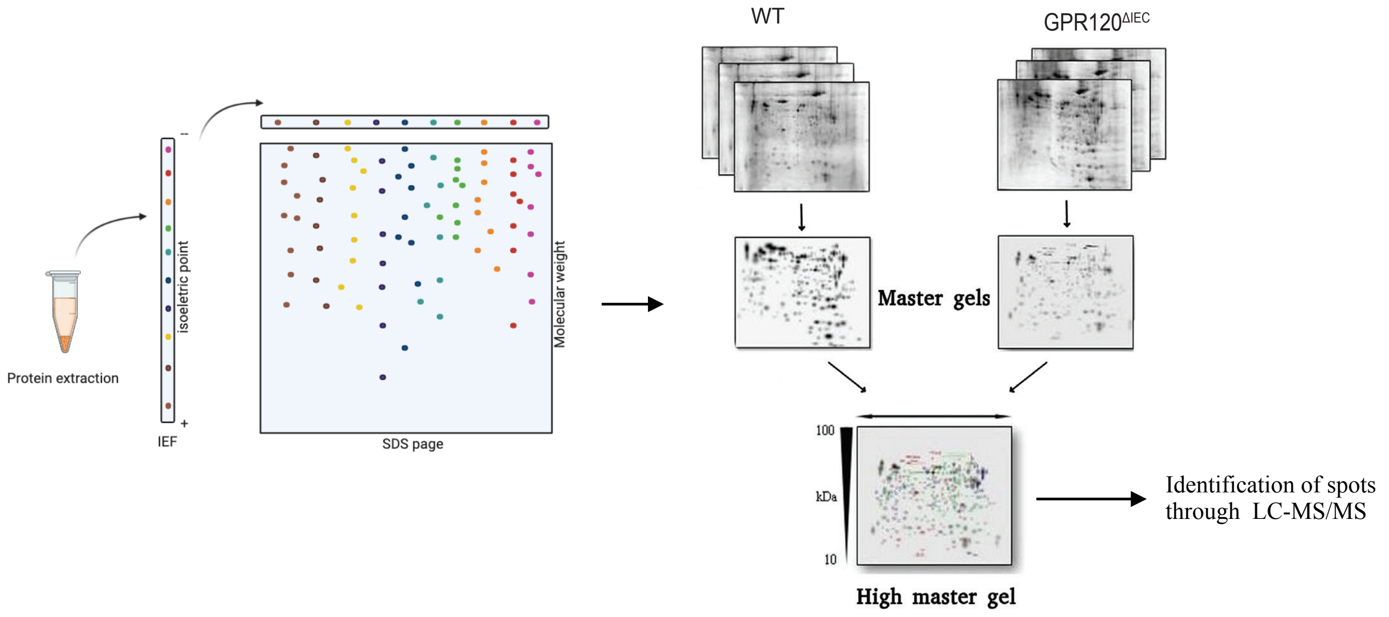

Supplement: Supplementary file 1 — Supplementary Information 1. [file 41598_2021_3787_MOESM1_ESM.pdf]
