# Supplementary figures and images for: GPR120 prevents colorectal adenocarcinoma progression by sustaining the mucosal barrier integrity
Source: Sci Rep. 2022 Jan 10;12:381. doi: 10.1038/s41598-021-03787-7 (PMC8748819; doi:10.1038/s41598-021-03787-7)

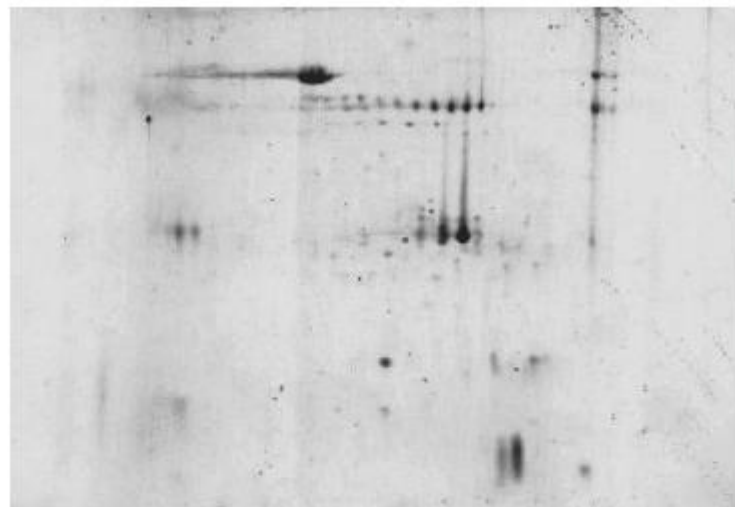

WT

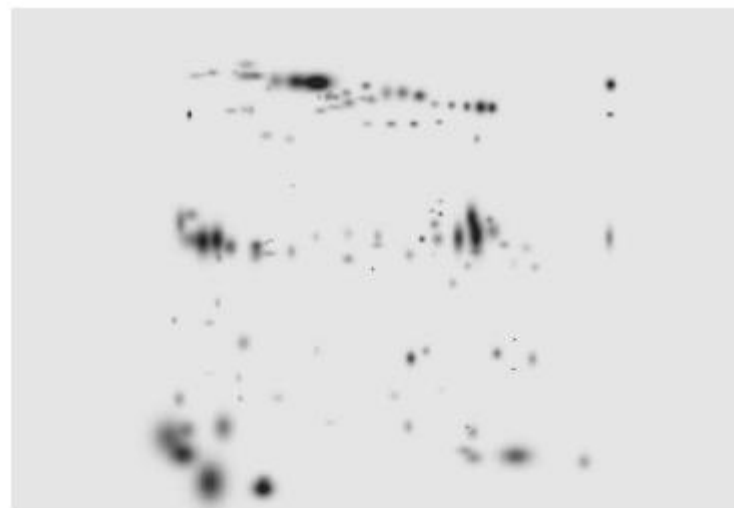

HMG

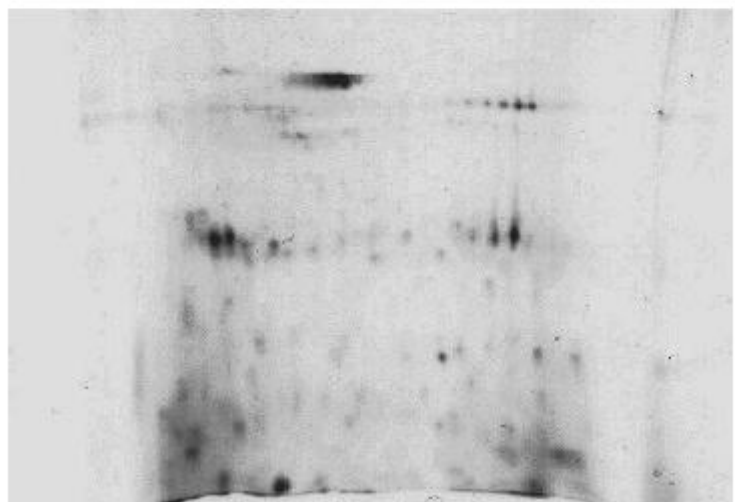

TG

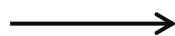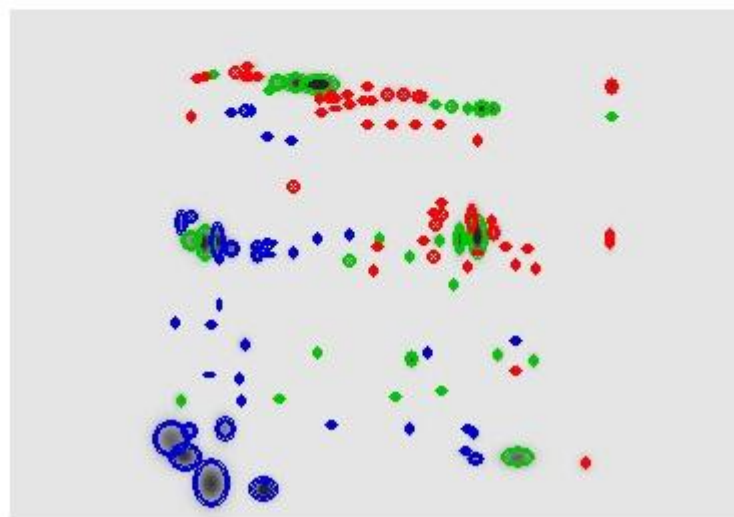

IN BOTH

TG

WT

Supplement: Supplementary file 2 — Supplementary Information 2. [file 41598_2021_3787_MOESM2_ESM.pdf]

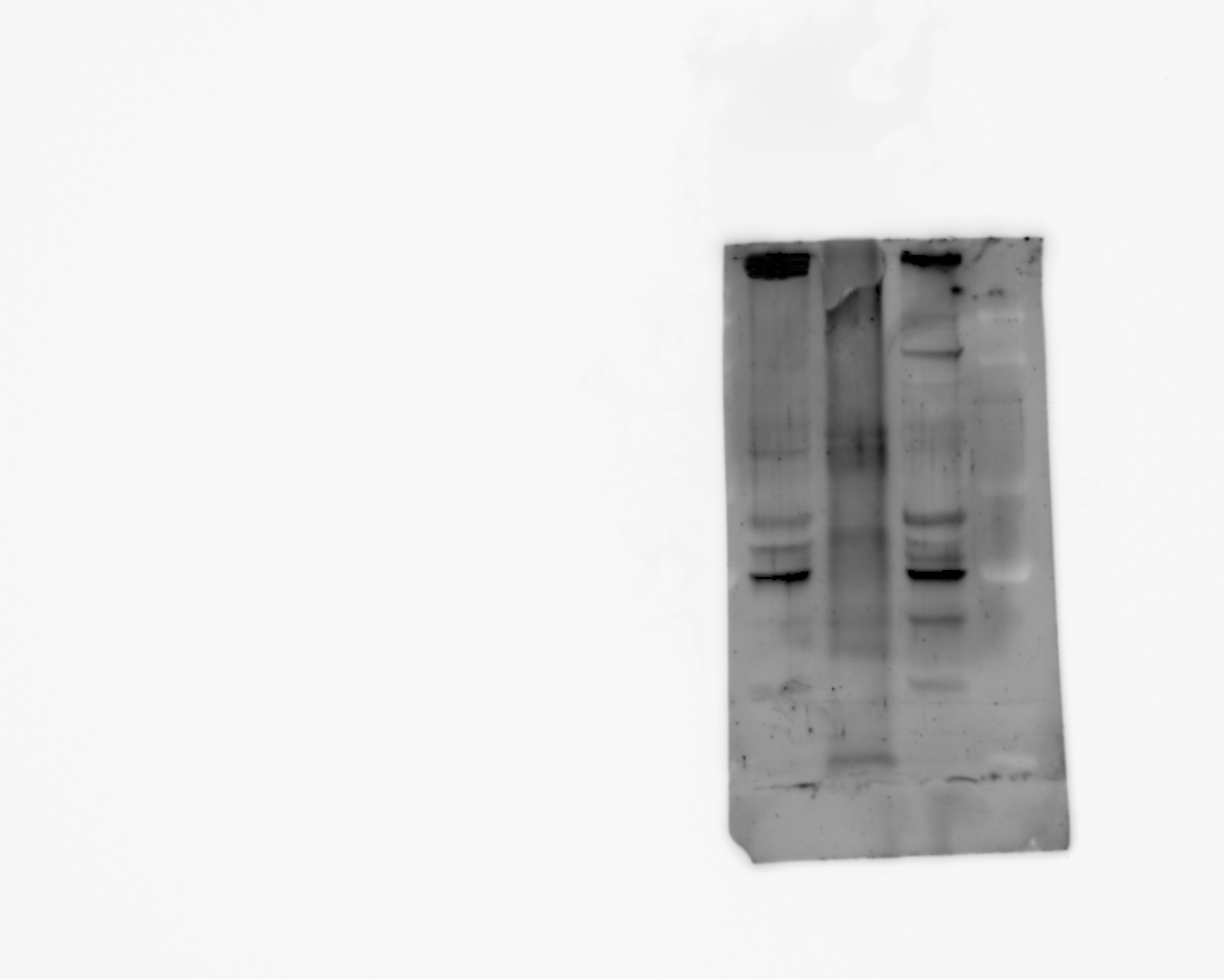

Supplement: Supplementary file 3 — Supplementary Information 3. [file 41598_2021_3787_MOESM3_ESM.tif]

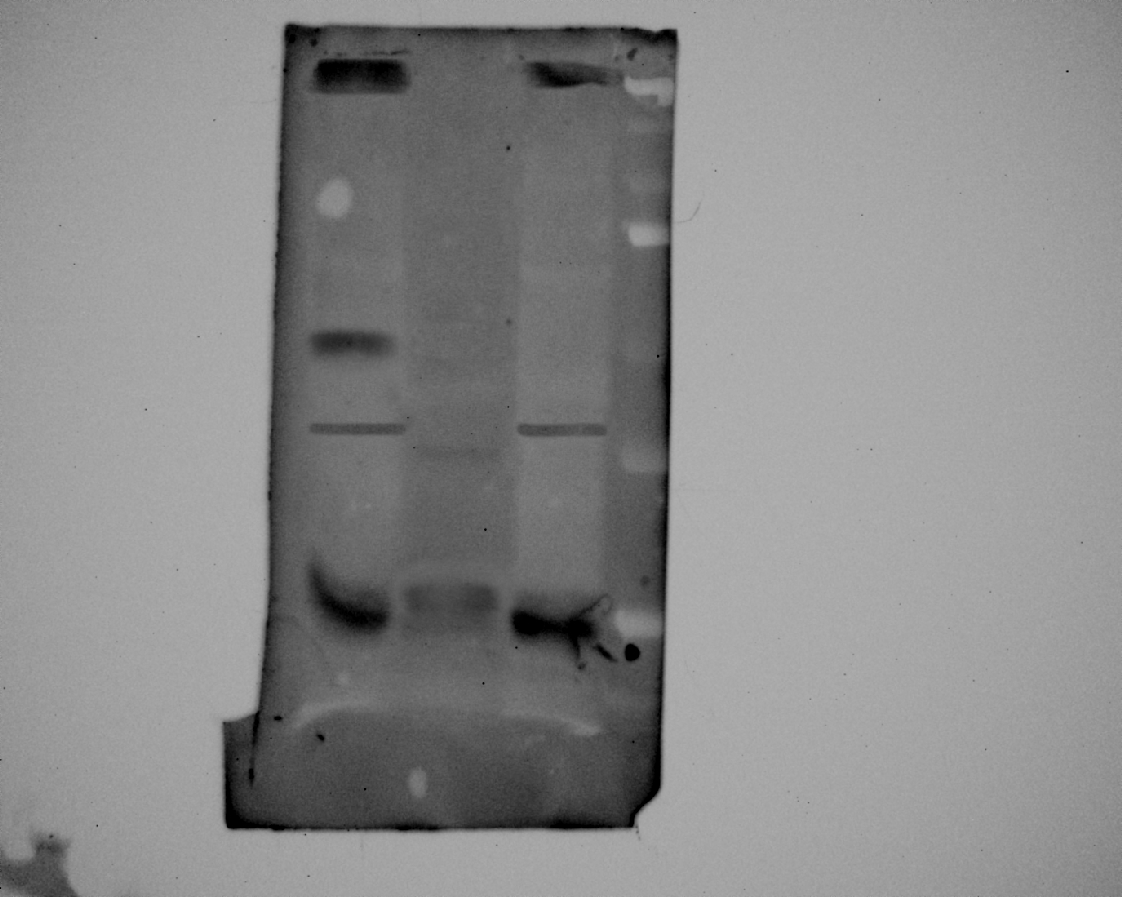

Supplement: Supplementary file 4 — Supplementary Information 4. [file 41598_2021_3787_MOESM4_ESM.tif]

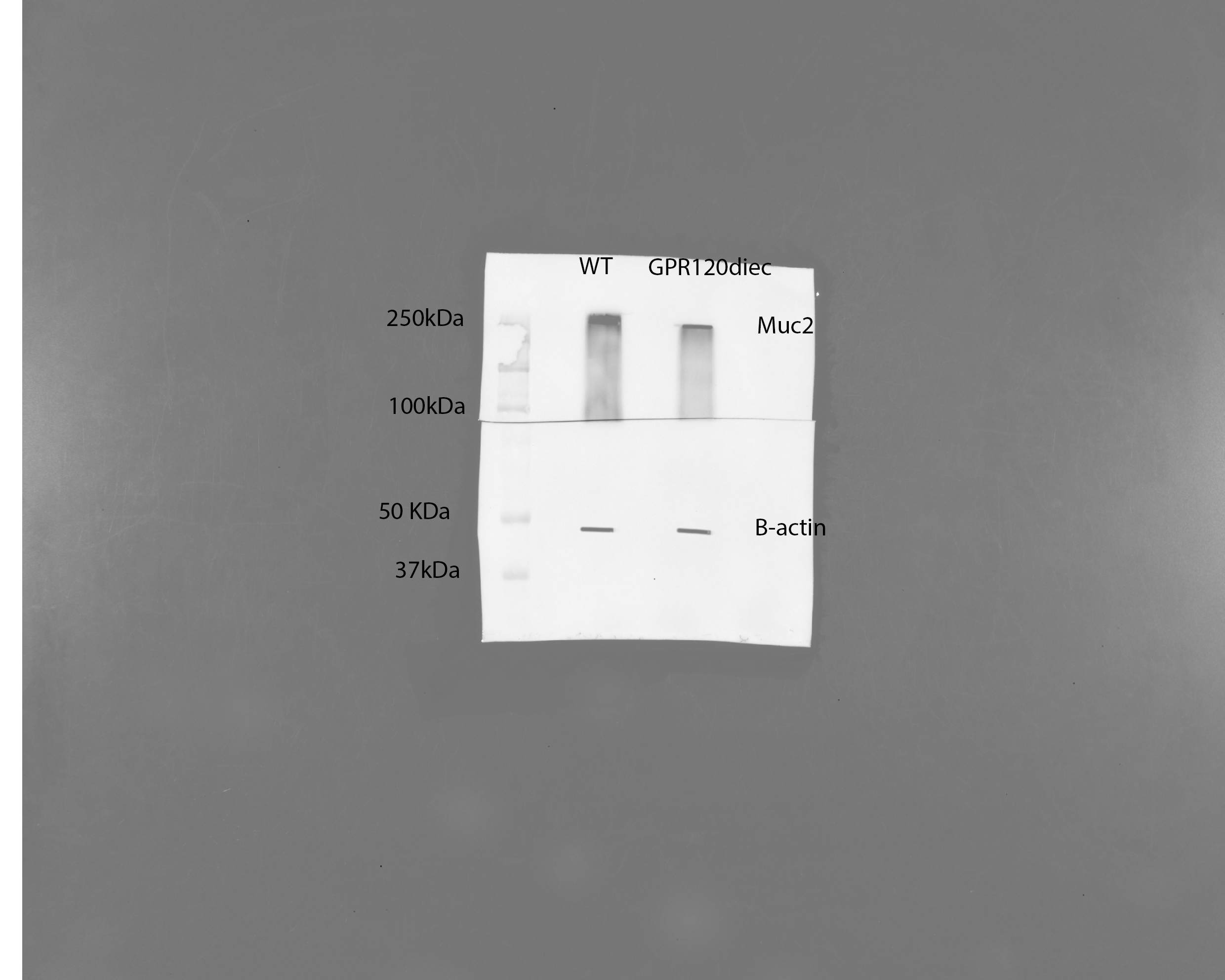

Supplement: Supplementary file 7 — Supplementary Information 7. [file 41598_2021_3787_MOESM7_ESM.tif]
