# Supplementary material for: GPR120 prevents colorectal adenocarcinoma progression by sustaining the mucosal barrier integrity
Source: Sci Rep. 2022 Jan 10;12:381. doi: 10.1038/s41598-021-03787-7 (PMC8748819; doi:10.1038/s41598-021-03787-7)
Supplement: Supplementary file 5 — Supplementary Information 5. [file 41598_2021_3787_MOESM5_ESM.doc]

**LacZ F1**

**LacZ R1**

**LacZ F**

**LacZ R**

**NeoF**

**NeoR**

**Ffar4 F**

**LAR3**

**Ffar4 R**

**FfarCreF**

**FfarCreF11**

**FfarCreR**

**FfarCreR 1**


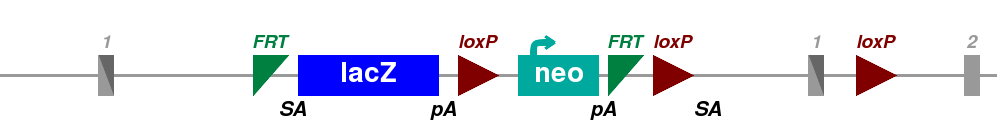


**LacZ F**: TTACTGCCGCCTGTTTTGAC

**LacZ R**: GACTGTAGCGGCTGATGTTG

Product size: 162 bp

**LacZ F1**: ACCGTTGATGTTGAAGTGGC

**LacZ R1**: GCCATAATTCAATTCGCGCG

Product size: 243 bp

**Ffar4 F**: CAAGTCAATCGCACCCACTT

**LAR3**: CAACGGGTTCTTCTGTTAGTCC

**Ffar4 R**: CGGCTTTGGTCAGATCCTTG

Product size: allele tg 397 bp; allele wt 589 bp

**FfarCre F**: TCAGTATCGGCGGAATTCC

**FfarCre R**: GAGCTCAGACCATAACTTCGT

Product size: 400 bp if between external loxP (1st and 3rd) everything has been removed; 1306bp if the region between 1st and 2nd LoxP has been removed; 2286bp if region between 2nd and 3rd LoxP has been removed; 3217bp if anything was removed.

**FfarCre F1**: GCGGAAGAAGGCACATGG

**FfarCre R1**: CTGAACTGATGGCGAGCTC

Product size: 485 bp if between external loxP (1st and 3rd) everything has been removed; 1398 bp if the region between 1st and 2nd LoxP has been removed; 2378 bp if region between 2nd and 3rd LoxP has been removed; 3309 bp if anything was removed

**NeoF**: GATGCTCTTCGTCCAGATCA

**NeoR**: CTCCTGCCGAGAAAGTATCC

Product size: 165 bp
