# Supplementary material for: GPR120 prevents colorectal adenocarcinoma progression by sustaining the mucosal barrier integrity
Source: Sci Rep. 2022 Jan 10;12:381. doi: 10.1038/s41598-021-03787-7 (PMC8748819; doi:10.1038/s41598-021-03787-7)
Supplement: Supplementary file 6 — Supplementary Information 6. [file 41598_2021_3787_MOESM6_ESM.docx]

**GPR120 prevents colorectal adenocarcinoma progression by sustaining the mucosal barrier integrity**

Federica Rubbino^1^, Valentina Garlatti^2^, Valeria Garzarelli^3^, Luca Massimino^4^, Salvatore Spanò^4^ , Paolo Iadarola^5^, Maddalena Cagnone^6^, Martin Giera^7^, Marieke Heijink^7^, Simone Guglielmetti^8^, Vincenzo Arena^9^, Alberto Malesci^4,10^, Luigi Laghi^10,11^, Silvio Danese^4,12^ & Stefania Vetrano*^4,12^

*Correspondence to: Stefania Vetrano, PhD

Assistant professor in Applied Biology

Humanitas University

Via Rita levi Montalcini

Pieve Emanuele (Milan) Italy

Stefania.vetrano@hunimed.eu

**Supplementary Figure 1**: (**a**) Representative immunostaining for GPR120 highlighting positive goblet cells (blue boxes) in mouse and human healthy colon (upper and lower panel, respectively). Scale bar: 50 μm. (**b**) Representative immunostaining for GPR120 in WT and GPR120^ΔIEC^ small intestine. Scale bar: 50 μm. (**c**) Relative transcriptome level of *FFAR4* (encoding for GPR120) in different cancer cell lines. (**d**) Caco-2 (**left panel**) and LoVo (**right panel**) cells were transfected with siRNA against *FFAR4* and scramble siRNA controls. After 48 hours from transfection, silencing efficacy was checked by qRT-PCR*.* (**e**) Heatmap shows genes related to Core Matrisome and Cell-Cell adhesion dataset (<https://www.gsea-msigdb.org/gsea/msigdb/cards/NABA_CORE_MATRISOME> ; <https://www.gsea-msigdb.org/gsea/msigdb/cards/KEGG_CELL_ADHESION_MOLECULES_CAMS> ) from RNA-seq performed on epithelial cells isolated from healthy WT and mutant mice (called here TG, n=3 for each group), selected by significant p.value (<0,05). (**f**) The expression levels of *Muc*2 gene was quantified by qRT-PCR in AOM/DSS GPR120^ΔIEC^(n=7) and WT (n=7) mice. Values are expressed as median ± 95% CI. **p.value<0,01 by Mann Whitney test.

**Supplementary Figure 2**: (**a**) OTUs distinguishing WT and GPR120 ^ΔIEC^ mice 8 weeks post-injection (T8) determined using the DESeq2 negative binomial distribution method on the 16S rRNA gene proﬁling data of fecal samples. The taxonomic lineage of each taxon is shown: p, phylum; c, class; o, order; f, family; g, genus; s, species. The black-yellow heatmap represents the mean normalized relative abundances of the reported OTUs. Positive fold changes (shown on a red background) designate OTU overrepresentation in GPR120 ^ΔIEC^ mice (KO); negative fold changes (shown on a blue background) designate the OTU overrepresentation in WT mice. padj, adjusted p values were represented in a heatmap. (**b, c**) Clinical parameters of intestinal inflammation in AOM/DSS protocol, such as body weight loss (**b**), and DAI score (**c**) were monitored. Light blue boxes indicate the recovery periods with drinking water. (**d**) Expression of pro-inflammatory cytokines by qRT-PCR evaluated in AOM/DSS treated mice (n=7 for each group). Values are expressed as median ± 95% CI.

**Supplementary Figure 3:** Representative images of paraffin-embedded tissue of T2 tumor immunostained against GPR120. The boxes draw attention to the positivity that is undetectable in the epithelium (box 1) and quantifiable in the immune infiltrate (box 2). Magnification: 20X. Scale bar: 50 μm.

**Supplementary Figure 4**: (**a, b**) Caco-2 and LoVo cells were stained with Annexin V/7-AAD 48 hours after transfection with either siRNA against *FFAR4* or scramble siRNA. The percentage of apoptotic cells over total singlets were quantified by FACS analysis. Values are expressed as median ± 95% CI of biological triplicates.

**Supplementary Figure 5**: (**a**) Omega-6 PUFAs were quantified and expressed as area ratio/feces concentration. (**b**) Gene expression levels of *Alox15* and *Alox15B* in samples of healthy (n=4 for each group) and AOM/DSS-treated GPR120^ΔIEC^and WT (n=7 for each group) littermates were quantified by qRT-PCR. Values are expressed as median ± 95% CI.

**Supplementary Figure 6:** Representative PCR bands from the mouse genotyping protocol. For *Ffar4* gene, the PCR products of two amplicon size 589bp and 397bp were generated and resolved on 2% agarose gel electrophoresis. A single band at 589bp and 397bp corresponded to WT and total KO (homo) respectively, whereas the double bands identified the heterozygous (het) mutant. For *VillinCre***,** transgenic mice were detected by PCR and products of different amplicon size were generated. Cre-recombinase insertion was identified by a single band at 3217 bp size.

**Supplementary Figure 7:** Schematic representation of 2-DE performed on mucus layer in which mucus was scraped off the epithelial layer, then samples were subjected to first separation depending on their isoelectric point, and then depending on their molecular weight. The gels obtained were overlaid in order to obtain a High master gel, used for identification of proteins by LC-MS/MS.

**Supplementary information**

The file “GPR120 muc2 blot.tif” is the entire membrane of the cropped image reported in the principal Figure 2b. The first line (from the left) is for WT sample, the other for the transgenic. Upper line is for MUC2 protein detection, the lower one for the housekeeping b-actin.

The file “GPR120_MUC2_duplicate.tif” is the biological duplicate used for the protein quantification reported in Figure 2b (lower panel). The first line (from the left) is for WT sample, the other for the transgenic. Upper line is for MUC2 protein detection, the lower one for the housekeeping b-actin.

The file “wb revisione3.tiff” is the biological triplicate used for the protein quantification reported in Figure 2b (lower panel). The membrane is cut in the middle (horizontally) due to antibodies’ species. As usual, the first line from the left is for WT sample, the other is for Transgenic.

The file “gel WTvsTG.pdf” is the full-size image (with evident membrane edge) of the image in Figure 2c.
